# Nitrogen‐Based Bisphosphonate Use and Risk of Cancer in Women Aged 50 Years and Older: A National Data Linkage Study

**DOI:** 10.1002/ijc.70535

**Published:** 2026-05-20

**Authors:** Karen M. Tuesley, Fiona Caristo, Katrina Spilsbury, Sallie‐Anne Pearson, Peter Donovan, Michael D. Coory, Christopher B. Steer, Susan J. Jordan

**Affiliations:** ^1^ School of Public Health, Faculty of Health Medicine & Behavioural Sciences University of Queensland Brisbane Australia; ^2^ Institute for Health Research The University of Notre Dame Australia Fremantle Australia; ^3^ School of Population Health University of New South Wales Sydney Australia; ^4^ Centre of Research Excellence in Medicines Intelligence University of New South Wales Sydney Australia; ^5^ Clinical Pharmacology Department Royal Brisbane and Women's Hospital Brisbane Australia; ^6^ Faculty of Health Medicine & Behavioural Sciences University of Queensland Brisbane Australia; ^7^ Mater Medical Research Institute University of Queensland Brisbane Australia; ^8^ Border Medical Oncology Albury‐Wodonga Regional Cancer Centre Albury Australia; ^9^ University of NSW Rural Clinical School Albury New South Wales Australia

**Keywords:** bisphosphonates, breast cancer, cancer, nested case–control, nitrogen‐based bisphosphonates

## Abstract

Bisphosphonates are used widely to treat osteoporosis and to manage bone metastases in patients with breast cancer. Some studies suggest that nitrogen‐based bisphosphonates (NBBs) may also reduce cancer risk more broadly, although evidence is inconsistent. Using linked administrative health data from Australian women aged > 50 years, we conducted nested case–control studies and used conditional logistic regression to estimate odds ratios (ORs) and 95% confidence intervals (CI) for the association between use of bisphosphonates (nitrogen‐based and other) and other medicines approved for treatment of low bone mineral density, and risk of breast, colorectal, lung, uterus, thyroid, pancreas, kidney, cervical, stomach, liver, brain, bladder and gallbladder cancers and melanoma. In total, 243,629 incident cancer cases were matched to 1,218,075 controls. Compared to no use of osteoporosis medicines, use of nitrogen‐based bisphosphonates (NBBs) was associated with reductions in breast (OR = 0.85, 95% CI: 0.83, 0.87), uterine (OR = 0.61, 95% CI: 0.57, 0.66) and cervical cancer risk (OR = 0.83, 95% CI: 0.71, 0.97) with the greatest reduction in risk observed for women who used NBBs for over 5 years. However, NBBs were also associated with increased risk of lung (OR = 1.16, 95% CI: 1.12, 1.21) and liver cancers (OR = 1.15, 95% CI: 1.02, 1.29), although there was no clear dose response. Raloxifene was also associated with lower risk of breast, colorectal, uterine and cervical cancer. Our exploratory study suggests links between some osteoporosis medicines and cancers related to reproductive hormones. Our results require confirmation but may provide new insights for cancer prevention.

## Abstract (highlights)

What's new?

Bisphosphonates are widely used to treat osteoporosis and to manage bone metastases in patients with breast cancer. Nitrogen‐based bisphosphonates may also broadly reduce cancer risk, but evidence remains inconsistent. This large population‐based data linkage study in women aged > 50 years found that, compared to no use of osteoporosis medicines, nitrogen‐based bisphosphonates were associated with reduced risk of breast, uterine, and cervical cancers, with the greatest risk reduction observed for use exceeding five years. Nitrogen‐based bisphosphonates were associated with an increased risk of lung and liver cancers. This exploratory study suggests links between osteoporosis medicines and cancers related to reproductive hormones.

## Graphical abstract

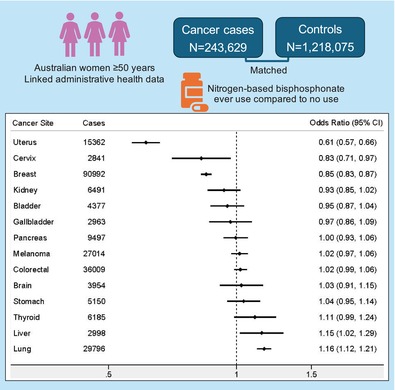

**Abbreviations**
ARIA: Accessibility/Remoteness Index of Australia
ATC: Anatomical Therapeutic Chemical
CI: confidence interval
DDD: defined daily dose
ICD‐O: International Classification of Disease for Oncology
MHT: menopausal hormone therapy
NBB: nitrogen‐based bisphosphonates
OR: odds ratio
PBS: Pharmaceutical Benefits Scheme
RCT: randomised controlled trial
RR: relative risk
SEIFA: Socio‐Economic Indexes for Areas
SES: socioeconomic status

## 1 Introduction

Bisphosphonate medicines have been used widely for osteoporotic fracture prevention for over 20 years and more recently they have been used to prevent and treat bone metastases in patients with breast or prostate cancer [1, 2]. Randomised controlled trials (RCTs) have shown that bisphosphonates improved overall survival and increased disease‐free survival in post‐menopausal women with early breast cancer when used in the adjuvant setting (albeit at doses higher than used for fracture prevention) [3]. It is therefore of interest whether bisphosphonates might play a role in reducing cancer incidence, but the evidence for this is not yet clear.

Preclinical studies have demonstrated plausible mechanisms for anti‐tumour effects of bisphosphonates beyond the skeleton [1]. Nitrogen‐based bisphosphonates (NBBs) in particular have been shown to induce cell apoptosis, inhibit tumour cell adhesion, disrupt tumour angiogenesis and alter polarisation of tumour‐associated macrophages (from growth promoting to inhibitory) [1], although it is not clear whether these could equate to meaningful effects on tumour development at the doses of bisphosphonate commonly used for osteoporotic fracture prevention.

Some evidence for cancer prevention benefits comes from a secondary analysis of an RCT of bisphosphonates for fracture prevention [4]. This study of zoledronic acid (zoledronate, an NBB) in 2000 women with low bone mineral density showed a reduction in cancer incidence after 6 years of follow‐up (RR = 0.69, 95% CI: 0.52, 0.89) that was largely driven by a reduction in breast cancer (RR = 0.59, 95% CI: 0.35, 0.98) [4]. However, a pooled analysis of another two RCTs investigating use of NBBs (alendronate, *n* = 6459 and zoledronic acid, *n* = 7765, respectively) found no reduction in breast cancer risk, although follow‐up time was shorter (mean follow‐up of 3.8 and 2.8 years, respectively) [5]. A meta‐analysis of observational studies assessing the association between bisphosphonate use and risk of any cancer type (19 different types in 34 studies) also reported a protective association between bisphosphonate use and risk of breast cancer (RR = 0.87, 95% CI: 0.54, 0.92), as well as inverse associations with endometrial (RR = 0.75, 95% CI: 0.54, 0.92) and colorectal cancers (RR = 0.89, 95% CI: 0.81, 0.98) [6]. They found no statistically significant associations with any of the other cancer types [6]; however, for most cancers there were relatively few studies and marked heterogeneity in the results. The included studies adjusted for a range of potential confounders but only four were restricted to participants with indications for bisphosphonates [7, 8, 9, 10]; a further nine adjusted for osteoporosis or hip fracture risk scores [11, 12, 13, 14, 15, 16, 17, 18, 19] and four considered confounding by indication in sensitivity analyses [20, 21, 22, 23].

Given the potential for anti‐tumour activity of bisphosphonates, suggestive observational data in humans (particularly for nitrogen‐based bisphosphonates) and the widespread use of bisphosphonate therapy for osteoporosis [24], it is important to clarify their potential relationship with cancer. Our group has previously used national linked data to investigate the association between use of NBBs and risk of ovarian cancer [25], finding a reduction in risk for ever use versus never use (OR = 0.81, 95% CI: 0.75, 0.88). Here we have expanded the analysis to assess the association between NBB use and incidence of the most common non‐haematopoietic cancers in women. We did not include haematopoietic cancers as those that occur most commonly in Australian women are mostly heterogenous malignancies in terms of aetiology and clinical presentation [26]. Our primary focus was on NBBs but we compared these with raloxifene and other osteoporosis medicines (denosumab, strontium, non‐nitrogen‐based bisphosphonates) in secondary analyses and conducted sensitivity analyses with respect to confounding by indication.

## 2 Methods

### 2.1 Study Design and Population

We conducted a series of nested case–control studies using linked administrative health data to investigate the association between NBB use and breast, colorectal, lung, uterus, thyroid, pancreas, kidney, cervical, stomach, liver, brain, bladder and gallbladder cancers and melanoma (study cancers). Cases and controls were selected from women living in Australia (aged 18 years or older) who had registered with Medicare, Australia's universal health insurance scheme before 1st July 2002. Medicine exposure, cancer diagnoses and deaths were determined using individual‐linked records from routinely collected national health data. Additional linkage to state‐based health data enabled adjustment for potential confounders.

### 2.2 Data Sources

For the overall cohort we included records from: (a) the Medicare Consumer Database including Medicare registration, residential postcode and year of birth; (b) the Pharmaceutical Benefits Scheme (PBS) with individual prescriptions dispensed under the government pharmaceutical subsidy scheme (from 1st July 2002 onwards); (c) the Australian Cancer Database including all newly diagnosed invasive primary cancers registered since 1st January 1982; and (d) the National Death Index with all deaths in Australia, including date, age and cause of death. We included additional data from Western Australia, Australia's fourth most populous state, from which we accessed records of: (a) current residential postcode (the Western Australian Electoral Roll); (b) inpatient diagnoses and procedures in Western Australia since 1970 (Hospital Morbidity Data); and (c) number of births (Birth Registrations [1950–1979], Midwives Notifications [1980 onwards]). The period covered by each data set is shown in Figure S1.

### 2.3 Eligibility

We required at least 2 years of PBS history before cancer diagnosis to ascertain medicine use, but excluded medicines dispensed within 6‐months of cancer diagnosis to avoid changes in medicine use related to pre‐diagnosis cancer symptoms. We used the International Classification of Disease for Oncology (ICD‐O) topography codes to identify the study cancers (Table S1). Cases included all those with a new diagnosis of one of the study cancers registered in the Australian Cancer Database between 1st July 2004 and 31st December 2013. We excluded women who were under 50 years at diagnosis (as bisphosphonate use is uncommon in this age group), those with a prior cancer diagnosis, and women who used bisphosphonates prescribed only for Paget's disease or cancer (pamidronate disodium, tiludronate, ibandronate and clodronate: Anatomical Therapeutic Chemical [ATC] codes M05BA02, M05BA03, M05BA05 and M05BA06).

**FIGURE 1:**
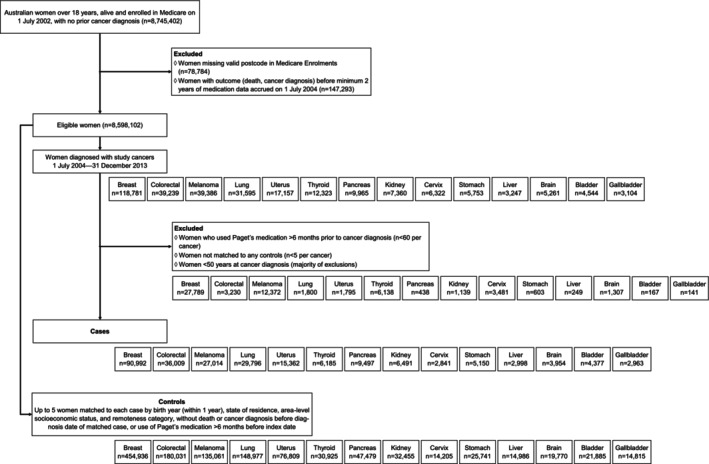
Selection of participants for each nested case–control analysis of nitrogen‐based bisphosphonate exposure and cancer incidence.

We defined the index date as the date of cancer diagnosis for each case. Up to five controls for each case were selected from all women in the study population who were alive, without a prior diagnosis of cancer, and had not been prescribed bisphosphonates for Paget's disease/cancer at the index date of each case. They were matched on birth year (within 1 year of case), state of residence, area‐level socioeconomic status (SES) and remoteness category. Women were eligible for selection as a control multiple times (and for different cancers), and women diagnosed with cancer were eligible to be selected as a control until their diagnosis date (Figure 1).

### 2.4 Exposure Variables

Osteoporosis medicine use was defined using the Anatomical Therapeutic Chemical (ATC) drug classification system and PBS item codes (Table S2), and included NBB, raloxifene, non‐nitrogen‐based bisphosphonates, strontium and denosumab. Participants were first classified as a user of a medicine if they had the medicine dispensed at least twice within a 12‐month period before their index date, except for the injectables zoledronic acid (an NBB) and denosumab, for which a woman was defined as a user after a single dispensed prescription. We classified women as exclusive users of NBB, raloxifene, non‐nitrogen‐based bisphosphonates or other osteoporosis medicines (strontium and/or denosumab), users of NBB plus one or more other types of osteoporosis medicine, users of more than one type of non‐NBB osteoporosis medicine, or never‐users of any osteoporosis medicines.

We calculated the duration of exclusive use of NBB from the total defined daily dose and the period covered by dispensed prescriptions. Total defined daily dose (DDD) for each woman was calculated from the DDD for each ATC code [27], the number of DDDs for each PBS item (Table S2) and the amount dispensed [25]. The period of use was the time (in years) between the date of supply of the first eligible prescription and the end of the last prescription (date of supply plus number of dosage days), or a woman's index date, whichever was the earlier. One year of NBB use was calculated as a period of use of 12 or more months (excluding the 6 months prior to index date) plus at least 80% [28] of 365.25 daily doses (DDD ≥ 292). Zoledronic acid for osteoporosis treatment is usually administered once per year [24], therefore we counted each injection as 1 year of use. We additionally defined women as users for 3 years if they had a period of use of at least 36 months and 80% of 3 years of daily doses (DDD ≥ 877), and users for 5 years or more if they had a period of use of at least 60 months and 80% of 5 years of daily doses (DDD ≥ 1461).

### 2.5 Covariates

We used residential postcodes to determine Australian state of residence at Medicare enrolment and assigned area‐level measures of SES and remoteness. Participants were categorised into SES quintiles using the Index of Relative Socio‐Economic Disadvantage scores from the Socio‐Economic Indexes for Areas (SEIFA) [29], with the highest quintile being those with least disadvantage. Women's area of residence was categorised as major city, inner regional, outer regional or remote/very remote, based on the Accessibility/Remoteness Index of Australia (ARIA) [30]. We excluded women who did not have a valid postcode to allow a SEIFA score to be assigned (< 1%; Figure 1).

To adjust for potential confounding by comorbid conditions, we used the validated weighted Rx‐Risk Comorbidity Score (Rx‐Risk score) [31] which has been mapped to PBS item codes [32]. While we did not have records for all medicines included in the Rx‐Risk score (Table S3) most of those missing had low or zero weighting or were used to treat rare conditions unlikely to materially affect the likelihood of a woman developing cancer. Medicines for depression and chronic airway disease were the only weighted, common medicines not included in the dataset. We additionally conducted a sensitivity analysis for women for whom we were confident that all PBS medicines were recorded and used other methods to assess the effects of adjusting for potential confounding by smoking (see sensitivity analyses below). We used the Rx‐Risk score for each woman calculated at 6‐months prior to the index date, with a maximum of 5‐years medicine history used to identify comorbidities for each woman. We excluded osteoporosis/Paget's disease from the Rx‐Risk score.

We defined women as menopausal hormone therapy (MHT) users if they were dispensed at least two of either oestrogen‐only or combined MHT (ATC codes G03C and G03F) within a 12‐month period at any point up to 6 months prior to the index date.

### 2.6 Statistical Analysis

We used conditional logistic regression to estimate odds ratios (ORs) and 95% confidence intervals (CI) for the association between NBB medicines and the risk of each cancer. The models were adjusted for the weighted Rx‐Risk score as a continuous variable. We assessed the association for exclusive use of NBB, raloxifene, non‐nitrogen‐based bisphosphonates, strontium and/or denosumab and NBBs combined with these other medicines, compared with women who had no history of using an osteoporosis medicine or raloxifene (since 1st July 2002) as the reference group. We then assessed the effect of duration of exclusive use of NBB use (less than 1 year, 1 year to less than 3 years, 3 years to less than 5 years, 5 or more years) compared to no use of NBB. In this last analysis we excluded cases diagnosed prior to 1st July 2008 to allow sufficient PBS history (5 years) to categorise women as users or non‐users. We used Stata 16.0 [33] for all analyses.

### 2.7 Sensitivity Analyses

We conducted a series of sensitivity analyses to assess the potential effect of unmeasured confounders and confounding by indication on our results. The first analyses were restricted to women from Western Australia, for whom we had additional records with information on some potential confounders. We used ICD codes from hospital records to identify women with a history of osteoporosis or fractures deemed most likely due to osteoporosis [34] (Table S4) between 10 years and 6 months before their index date. We also identified women who had a record of gynaecological surgical procedures, including hysterectomy and oophorectomy more than 6 months prior to index date (Table S5). We calculated parity using birth and midwife records and categorised the number of births as 0, 1, 2, 3, or 4+. We adjusted specific analyses for these potential confounders accordingly. For our analysis of uterine and cervical cancer, we excluded women with hysterectomy prior to index date from selection as controls, as they were no longer at risk of these cancers.

In our second set of sensitivity analyses we restricted our analyses to the group of women for whom we were confident that all PBS medicines were recorded (including cheaper medicines, such as some types of MHT, that are not subsidised for general beneficiaries; see Supporting Information Methods for PBS concessional eligibility criteria). In this group we adjusted our analysis of NBB use and risk of hormone‐related cancers (endometrial, cervical, breast and colorectal cancers) for use of MHT. We also adjusted analyses for each cancer type by use of type II diabetes medicine (ATC codes A10BA01–A10BX99, excludes use of insulin alone) as a proxy measure for obesity [35]. When adjusting for diabetes medicine, we removed diabetes mellitus from the Rx‐Risk Score.

Finally, we used quantitative bias analysis [36] to quantify the potential effect of unmeasured confounders, focusing on two specific relationships. Firstly, we assessed the effects of adjusting for obesity on our analyses of the association between NBB and uterine cancer; second, we assessed the effects of adjusting for smoking on our analyses of the association between NBB and lung cancer. We used available data (see Supporting Information Methods) for the association between the confounders (obesity, smoking) and the exposure (NBB use), and the confounders and the outcomes (uterine cancer, lung cancer).

## 3 Results

### 3.1 Study Population

**FIGURE 2:**
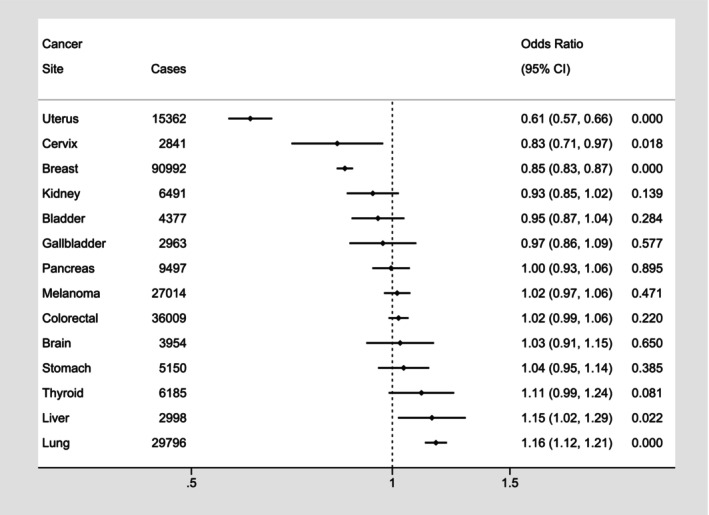
Odds ratios for ever‐use of nitrogen‐based bisphosphonate and incidence of cancer, adjusted for comorbidity (weighted Rx‐Risk score), in Australian women diagnosed at 50 years or older (Forest plot). CI, confidence interval.

In the study period we identified 243,629 incident cases of the study cancers in women aged 50 years and older who had not had a previous cancer diagnosis. These were matched with 1,218,075 controls who were alive and without a cancer diagnosis at the index date. Table 1 shows the number of women with each cancer and the distribution of demographic variables used for matching. Detailed data on the proportion of cases and controls by cancer who used osteoporosis medicines, MHT and diabetes medicines, as well as mean weighted Rx‐Risk Scores, are shown in Table 2. ORs for the association between NBB and raloxifene use and risk of study cancers are presented in Table 3 and Figure 2 (NBB only), with adjustment for weighted Rx‐Risk Scores. Associations for other osteoporosis medicines are shown in Table S6. Associations by duration of NBB use and each cancer type are presented in Table 4, while the results of our sensitivity analyses are included in Tables S7–S18. Results for each cancer are described below.

**TABLE 1:** Characteristics of women diagnosed with cancer, by matching variables, national data.

Characteristic	Cancer type													
	Breast	Colorectal	Melanoma	Lung	Uterus	Thyroid	Pancreas	Kidney	Cervix	Stomach	Liver	Brain	Bladder	Gallbladder
Total cases	*N* = 90,992	*N* = 36,009	*N* = 27014	*N* = 29,796	*N* = 15,362	*N* = 6185	*N* = 9497	*N* = 6491	*N* = 2841	*N* = 5150	*N* = 2998	*N* = 3954	*N* = 4377	*N* = 2963
Index age (years), mean (SD)	66.2 (10.9)	73.6 (11.0)	68.5 (11.6)	71.6 (10.4)	66.8 (10.2)	62.9 (9.9)	75.0 (11.0)	69.8 (11.2)	66.4 (11.7)	74.2 (11.4)	73.2 (11.4)	69.3 (10.8)	77.1 (10.6)	74.9 (10.8)
Index age (years)														
50–59	30,712 (34%)	4913 (14%)	7844 (29%)	4674 (16%)	4475 (29%)	2904 (47%)	1030 (11%)	1486 (23%)	1055 (37%)	729 (14%)	488 (16%)	943 (24%)	324 (7%)	328 (11%)
60–69	30,387 (33%)	8376 (23%)	7732 (29%)	8606 (29%)	5586 (36%)	1862 (30%)	2116 (22%)	1854 (29%)	759 (27%)	1097 (21%)	634 (21%)	1147 (29%)	810 (19%)	632 (21%)
70–79	17,356 (19%)	11,076 (31%)	6098 (23%)	9327 (31%)	3327 (22%)	959 (16%)	2787 (29%)	1766 (27%)	557 (20%)	1508 (29%)	895 (30%)	1096 (28%)	1260 (29%)	939 (32%)
80–89	10,361 (11%)	9778 (27%)	4377 (16%)	6254 (21%)	1712 (11%)	405 (7%)	2855 (30%)	1174 (18%)	391 (14%)	1456 (28%)	822 (27%)	670 (17%)	1523 (35%)	869 (29%)
≥ 90	2176 (2%)	1866 (5%)	963 (4%)	935 (3%)	262 (2%)	55 (1%)	709 (7%)	211 (3%)	79 (3%)	360 (7%)	159 (5%)	98 (2%)	460 (11%)	195 (7%)
State of residence at Medicare enrolment														
NSW	30,320 (33%)	12,089 (34%)	9441 (35%)	10,242 (34%)	5042 (33%)	2474 (40%)	3404 (36%)	2170 (33%)	1012 (36%)	1747 (34%)	1137 (38%)	1235 (31%)	1501 (34%)	904 (31%)
ACT	1505 (2%)	455 (1%)	343 (1%)	318 (1%)	225 (1%)	87 (1%)	94 (1%)	86 (1%)	28 (1%)	39 (1%)	31 (1%)	48 (1%)	32 (1%)	32 (1%)
VIC	22,622 (25%)	9057 (25%)	5772 (21%)	7377 (25%)	4055 (26%)	1268 (21%)	2449 (26%)	1642 (25%)	648 (23%)	1469 (29%)	797 (27%)	1149 (29%)	1043 (24%)	839 (28%)
QLD	17,501 (19%)	7074 (20%)	6519 (24%)	5538 (19%)	2970 (19%)	1302 (21%)	1581 (17%)	1238 (19%)	600 (21%)	822 (16%)	482 (16%)	664 (17%)	839 (19%)	524 (18%)
SA/NT	8174 (9%)	3237 (9%)	1904 (7%)	2555 (9%)	1489 (10%)	339 (5%)	822 (9%)	591 (9%)	210 (7%)	484 (9%)	246 (8%)	359 (9%)	432 (10%)	296 (10%)
WA	8568 (9%)	2999 (8%)	2352 (9%)	2851 (10%)	1224 (8%)	599 (10%)	884 (9%)	578 (9%)	256 (9%)	436 (8%)	240 (8%)	380 (10%)	378 (9%)	283 (10%)
TAS	2302 (3%)	1098 (3%)	683 (3%)	915 (3%)	357 (2%)	116 (2%)	263 (3%)	186 (3%)	87 (3%)	153 (3%)	65 (2%)	119 (3%)	152 (3%)	85 (3%)
SEIFA														
1 (most disadvantaged)	16,841 (19%)	7315 (20%)	4719 (17%)	7274 (24%)	3295 (21%)	1295 (21%)	2084 (22%)	1462 (23%)	739 (26%)	1211 (24%)	792 (26%)	746 (19%)	957 (22%)	652 (22%)
2	18,342 (20%)	7782 (22%)	6070 (22%)	6749 (23%)	3125 (20%)	1192 (19%)	1934 (20%)	1420 (22%)	643 (23%)	1034 (20%)	579 (19%)	852 (22%)	1002 (23%)	664 (22%)
3	17,994 (20%)	7269 (20%)	5488 (20%)	5948 (20%)	3050 (20%)	1188 (19%)	1917 (20%)	1343 (21%)	540 (19%)	1002 (19%)	575 (19%)	796 (20%)	816 (19%)	567 (19%)
4	18,119 (20%)	6877 (19%)	5244 (19%)	5117 (17%)	2994 (19%)	1186 (19%)	1811 (19%)	1171 (18%)	473 (17%)	1012 (20%)	546 (18%)	763 (19%)	822 (19%)	574 (19%)
5 (least disadvantaged)	19,696 (22%)	6766 (19%)	5493 (20%)	4708 (16%)	2898 (19%)	1324 (21%)	1751 (18%)	1095 (17%)	446 (16%)	891 (17%)	506 (17%)	797 (20%)	780 (18%)	506 (17%)
Remoteness														
Major City	62,536 (69%)	23,520 (65%)	17,187 (64%)	19,840 (67%)	10,417 (68%)	4514 (73%)	6482 (68%)	4327 (67%)	1921 (68%)	3683 (72%)	2222 (74%)	2711 (69%)	2948 (67%)	2012 (68%)
Inner Regional	19,480 (21%)	8518 (24%)	6761 (25%)	6700 (22%)	3280 (21%)	1100 (18%)	2042 (22%)	1514 (23%)	595 (21%)	991 (19%)	503 (17%)	858 (22%)	977 (22%)	639 (22%)
Outer Regional	7812 (9%)	3507 (10%)	2723 (10%)	2787 (9%)	1416 (9%)	469 (8%)	872 (9%)	563 (9%)	269 (9%)	423 (8%)	225 (8%)	342 (9%)	403 (9%)	253 (9%)
Remote & Very Remote	1164 (1%)	464 (1%)	343 (1%)	469 (2%)	249 (2%)	102 (2%)	101 (1%)	87 (1%)	56 (2%)	53 (1%)	48 (2%)	43 (1%)	49 (1%)	59 (2%)

Abbreviations: SD, standard deviation; SEIFA, Socio‐Economic Indexes for Areas.

**TABLE 2:** Descriptive statistics for eligible cases and controls of each study cancer by medicine use.

Category	Breast cancer		Colorectal cancer		Melanoma		Lung cancer		Uterine cancer	
	Case	Control	Case	Control	Case	Control	Case	Control	Case	Control
Total	*N* = 90,992	*N* = 454,936	*N* = 36,009	*N* = 180,031	*N* = 27,014	*N* = 135,061	*N* = 29,796	*N* = 148,977	*N* = 15,362	*N* = 76,809
Osteoporosis medicines^a^										
Non‐user	82,550 (91%)	405,786 (89%)	29,727 (83%)	148,917 (83%)	23,546 (87%)	117,782 (87%)	24,277 (81%)	125,679 (84%)	14,261 (93%)	68,446 (89%)
NBB only	6934 (8%)	39,347 (9%)	5075 (14%)	24,693 (14%)	2808 (10%)	13,781 (10%)	4384 (15%)	18,467 (12%)	874 (6%)	6611 (9%)
Raloxifene only	408 (0%)	2988 (1%)	367 (1%)	2054 (1%)	210 (1%)	1039 (1%)	345 (1%)	1432 (1%)	86 (1%)	515 (1%)
nNBB only	73 (0%)	403 (0%)	59 (0%)	272 (0%)	23 (0%)	146 (0%)	39 (0%)	181 (0%)	< 6	74 (0%)
Strontium and/or denosumab only	272 (0%)	1487 (0%)	159 (0%)	816 (0%)	94 (0%)	497 (0%)	131 (0%)	704 (0%)	< 35	303 (0%)
NBB and other osteo medicine/s	734 (1%)	4759 (1%)	606 (2%)	3163 (2%)	324 (1%)	1733 (1%)	595 (2%)	2422 (2%)	102 (1%)	832 (1%)
> 1 osteo medicine, excluding NBB	21 (0%)	166 (0%)	16 (0%)	116 (0%)	9 (0%)	83 (0%)	25 (0%)	92 (0%)	0 (0%)	28 (0%)
Weighted Rx‐Risk Score (mean (SD))	0.5 (2.3)	0.5 (2.3)	1.0 (2.7)	0.9 (2.7)	0.7 (2.5)	0.7 (2.4)	1.5 (2.9)	0.8 (2.6)	0.5 (2.4)	0.6 (2.3)
Diabetes (per Rx‐Risk)	7658 (8%)	36,319 (8%)	4077 (11%)	17,982 (10%)	2115 (8%)	11,363 (8%)	3217 (11%)	15,146 (10%)	2234 (15%)	6810 (9%)
MHT^b^										
Non‐user	72,387 (80%)	372,841 (82%)	29,194 (81%)	143,919 (80%)	21,592 (80%)	109,569 (81%)	23,728 (80%)	117,918 (79%)	13,332 (87%)	61,940 (81%)
Oestrogen only	11,092 (12%)	57,533 (13%)	5021 (14%)	27,642 (15%)	3996 (15%)	18,441 (14%)	4206 (14%)	23,211 (16%)	1321 (9%)	10,473 (14%)
Oestrogen & progestogen	7513 (8%)	24,562 (5%)	1794 (5%)	8470 (5%)	1426 (5%)	7051 (5%)	1862 (6%)	7848 (5%)	709 (5%)	4396 (6%)

Category	Thyroid cancer		Pancreatic cancer		Kidney cancer		Cervical cancer		Stomach cancer	
	Case	Control	Case	Control	Case	Case	Control	Case	Control	Case
Total	*N* = 6185	*N* = 30,925	*N* = 9497	*N* = 47,479	*N* = 6491	*N* = 6185	*N* = 30,925	*N* = 9497	*N* = 47,479	*N* = 6491
Osteoporosis medicines^a^										
Non‐user	5642 (91%)	28,392 (92%)	7658 (81%)	38,492 (81%)	5589 (86%)	27,832 (86%)	2577 (91%)	12,648 (89%)	4195 (81%)	21,189 (82%)
NBB only	443 (7%)	2000 (6%)	1478 (16%)	7133 (15%)	720 (11%)	3668 (11%)	215 (8%)	1232 (9%)	775 (15%)	3677 (14%)
Raloxifene only	26 (0%)	146 (0%)	97 (1%)	541 (1%)	60 (1%)	285 (1%)	15 (1%)	97 (1%)	49 (1%)	293 (1%)
nNBB only	< 6	18 (0%)	< 15	93 (0%)	< 6	38 (0%)	< 6	19 (0%)	< 10	34 (0%)
Strontium and/or denosumab only	11 (0%)	84 (0%)	52 (1%)	277 (1%)	20 (0%)	133 (0%)	< 10	46 (0%)	23 (0%)	110 (0%)
NBB and other osteo medicine/s	58 (1%)	278 (1%)	193 (2%)	903 (2%)	97 (1%)	478 (1%)	27 (1%)	153 (1%)	100 (2%)	424 (2%)
> 1 osteo medicine, excluding NBB	< 6	7 (0%)	< 6	40 (0%)	< 6	21 (0%)	0 (0%)	10 (0%)	< 6	14 (0%)
Weighted Rx‐Risk Score (mean (SD))	0.5 (2.2)	0.4 (2.1)	1.4 (2.9)	1.0 (2.8)	1.0 (2.8)	0.7 (2.5)	0.7 (2.3)	0.6 (2.3)	1.3 (2.9)	1.0 (2.7)
Diabetes (per Rx‐Risk)	561 (9%)	2309 (7%)	1765 (19%)	4925 (10%)	949 (15%)	3055 (9%)	285 (10%)	1166 (8%)	743 (14%)	2756 (11%)
MHT^b^										
Non‐user	5073 (82%)	26,036 (84%)	7574 (80%)	38,010 (80%)	5131 (79%)	26,021 (80%)	2583 (91%)	11,746 (83%)	4249 (83%)	20,846 (81%)
Oestrogen only	775 (13%)	3238 (10%)	1497 (16%)	7378 (16%)	1004 (15%)	4701 (14%)	151 (5%)	1741 (12%)	701 (14%)	3761 (15%)
Oestrogen & progestogen	337 (5%)	1651 (5%)	426 (4%)	2091 (4%)	356 (5%)	1733 (5%)	107 (4%)	718 (5%)	200 (4%)	1134 (4%)

Category	Liver cancer		Brain cancer		Bladder cancer		Gallbladder cancer	
	Case	Control	Case	Control	Case	Case	Control	Case
Total	*N* = 2998	*N* = 14,986	*N* = 3954	*N* = 19,770	*N* = 4377	*N* = 21,885	*N* = 2963	*N* = 14,815
Osteoporosis medicines^a^								
Non‐user	2402 (80%)	12,407 (83%)	3433 (87%)	17,152 (87%)	3455 (79%)	17,304 (79%)	2407 (81%)	12,004 (81%)
NBB only	474 (16%)	2034 (14%)	423 (11%)	2061 (10%)	730 (17%)	3729 (17%)	450 (15%)	2236 (15%)
Raloxifene only	40 (1%)	190 (1%)	26 (1%)	163 (1%)	62 (1%)	294 (1%)	37 (1%)	171 (1%)
nNBB only	< 6	23 (0%)	< 6	27 (0%)	< 15	40 (0%)	< 6	23 (0%)
Strontium and/or denosumab only	23 (1%)	74 (0%)	< 15	70 (0%)	17 (0%)	104 (0%)	16 (1%)	83 (1%)
NBB and other osteo medicine/s	51 (2%)	248 (2%)	57 (1%)	289 (1%)	101 (2%)	391 (2%)	47 (2%)	289 (2%)
> 1 osteo medicine, excluding NBB	< 6	10 (0%)	< 6	8 (0%)	< 6	23 (0%)	< 6	9 (0%)
Weighted Rx‐Risk Score (mean (SD))	1.5 (2.9)	1.0 (2.7)	0.7 (2.5)	0.7 (2.5)	1.4 (3.0)	1.2 (2.8)	1.3 (2.9)	1.0 (2.7)
Diabetes (per Rx‐Risk)	697 (23%)	1644 (11%)	341 (9%)	1835 (9%)	560 (13%)	2342 (11%)	467 (16%)	1600 (11%)
MHT^b^								
Non‐user	2432 (81%)	12,092 (81%)	3201 (81%)	16,006 (81%)	3506 (80%)	17,591 (80%)	2438 (82%)	11,882 (80%)
Oestrogen only	435 (15%)	2211 (15%)	562 (14%)	2726 (14%)	676 (15%)	3418 (16%)	406 (14%)	2274 (15%)
Oestrogen & progestogen	131 (4%)	683 (5%)	191 (5%)	1038 (5%)	195 (4%)	876 (4%)	119 (4%)	659 (4%)

Abbreviations: NBB, nitrogen‐based bisphosphonates; nNBB, non‐nitrogen‐based bisphosphonates; PBS, Pharmaceutical Benefits Scheme; SD, standard deviation.

^a^ Exclusive use of each osteoporosis medicine type or combined medicines.

^b^ Menopause Hormone Therapy, use = 2 or more scripts in 12 months.

**TABLE 3:** Association of nitrogen‐based bisphosphonate and raloxifene use with cancers in women.

Cancer site	Osteoporosis medicine	Cases		Controls		Adjusted model^a^	
		*n*	%	*n*	%	OR	95% CI
Breast	No use	82,550	91	405,786	89	Reference	
	NBB^b^	6934	8	39,347	9	0.85	0.83, 0.87
	Raloxifene^b^	408	0	2988	1	0.66	0.59, 0.73
Colorectal	No use	29,727	83	148,917	83	Reference	
	NBB^b^	5075	14	24,693	14	1.02	0.99, 1.06
	Raloxifene^b^	367	1	2054	1	0.89	0.79, 0.99
Melanoma	No use	23,546	87	117,782	87	Reference	
	NBB^b^	2808	10	13,781	10	1.02	0.97, 1.06
	Raloxifene^b^	210	1	1039	1	1.01	0.87, 1.17
Lung	No use	24,277	81	125,679	84	Reference	
	NBB^b^	4384	15	18,467	12	1.16	1.12, 1.21
	Raloxifene^b^	345	1	1432	1	1.18	1.05, 1.33
Uterus	No use	14,261	93	68,446	89	Reference	
	NBB^b^	874	6	6611	9	0.61	0.57, 0.66
	Raloxifene^b^	86	1	515	1	0.77	0.61, 0.97
Thyroid	No use	5642	91	28,392	92	Reference	
	NBB^b^	443	7	2000	6	1.11	0.99, 1.24
	Raloxifene^b^	26	0	146	0	0.90	0.59, 1.37
Pancreas	No use	7658	81	38,492	81	Reference	
	NBB^b^	1478	16	7133	15	1.00	0.93, 1.06
	Raloxifene^b^	97	1	541	1	0.87	0.70, 1.08
Kidney	No use	5589	86	27,832	86	Reference	
	NBB^b^	720	11	3668	11	0.93	0.85, 1.02
	Raloxifene^b^	60	1	285	1	1.02	0.77, 1.34
Cervix	No use	2577	91	12,648	89	Reference	
	NBB^b^	215	8	1232	9	0.83	0.71, 0.97
	Raloxifene^b^	15	1	97	1	0.74	0.43, 1.27
Stomach	No use	4195	81	21,189	82	Reference	
	NBB^b^	775	15	3677	14	1.04	0.95, 1.14
	Raloxifene^b^	49	1	293	1	0.83	0.61, 1.13
Liver	No use	2402	80	12,407	83	Reference	
	NBB^b^	474	16	2034	14	1.15	1.02, 1.29
	Raloxifene^b^	40	1	190	1	1.02	0.72, 1.44
Brain	No use	3433	87	17,152	87	Reference	
	NBB^b^	423	11	2061	10	1.03	0.91, 1.15
	Raloxifene^b^	26	1	163	1	0.80	0.53, 1.21
Bladder	No use	3455	79	17,304	79	Reference	
	NBB^b^	730	17	3729	17	0.95	0.87, 1.04
	Raloxifene^b^	62	1	294	1	1.03	0.78, 1.36
Gallbladder	No use	2407	81	12,004	81	Reference	
	NBB^b^	450	15	2236	15	0.97	0.86, 1.09
	Raloxifene^b^	37	1	171	1	1.03	0.72, 1.47

Abbreviations: CI: confidence interval; NBB, nitrogen‐based bisphosphonates; OR, odds ratio.

^a^ Models adjusted for weighted Rx‐Risk score, and matched by age, SEIFA, remoteness and registered state.

^b^ Exclusive use of either NBB or raloxifene, no use of other osteoporosis medicines.

**TABLE 4:** Duration of nitrogen‐based bisphosphonate use and incidence of cancers in women with at least 5 years of dispensing history.

Cancer site^a^	Osteoporosis medicine	Cases		Controls		Total	Adjusted model^b^		Trend with ‘no use’	Trend without ‘no use’
		*n*	%	*n*	%	*n*	OR	95% CI	*p* ^c^	*p* ^c^
Breast	No use	50,844	90	249,829	88	300,673	Reference		< 0.001	0.329
	< 1 year NBB use^d^	1077	2	5739	2	6816	0.90	0.85, 0.97		
	1 to < 3 years NBB use^d^, ^e^	1114	2	6227	2	7341	0.86	0.81, 0.92		
	3 to < 5 years NBB use^d^, ^f^	908	2	5341	2	6249	0.82	0.76, 0.88		
	≥ 5 years NBB use^d^, ^g^	1463	3	8459	3	9922	0.83	0.78, 0.88		
Colorectal	No use	17,317	81	86,344	81	103,661	Reference		0.415	0.147
	< 1 year NBB use^d^	674	3	3322	3	3996	1.00	0.92, 1.09		
	1 to < 3 years NBB use^d^, ^e^	770	4	3726	3	4496	1.02	0.94, 1.10		
	3 to < 5 years NBB use^d^, ^f^	653	3	3244	3	3897	0.99	0.91, 1.08		
	≥ 5 years NBB use^d^, ^g^	1113	5	5660	5	6773	0.97	0.90, 1.04		
Melanoma	No use	14,250	86	71,068	86	85,318	Reference		0.886	0.787
	< 1 year NBB use^d^	394	2	1937	2	2331	1.01	0.90, 1.12		
	1 to < 3 years NBB use^d^, ^e^	432	3	2211	3	2643	0.97	0.87, 1.07		
	3 to < 5 years NBB use^d^, ^f^	379	2	1917	2	2296	0.98	0.87, 1.09		
	≥ 5 years NBB use^d^, ^g^	633	4	3126	4	3759	1.00	0.91, 1.09		
Lung	No use	14,620	79	76,242	83	90,862	Reference		< 0.001	0.067
	< 1 year NBB use^d^	663	4	2507	3	3170	1.30	1.19, 1.43		
	1 to < 3 years NBB use^d^, ^e^	780	4	2902	3	3682	1.31	1.21, 1.43		
	3 to < 5 years NBB use^d^, ^f^	589	3	2563	3	3152	1.15	1.05, 1.27		
	≥ 5 years NBB use^d^, ^g^	954	5	4243	5	5197	1.13	1.05, 1.22		
Uterus	No use	8960	92	42,782	88	51,742	Reference		< 0.001	0.021
	< 1 year NBB use^d^	133	1	978	2	1111	0.62	0.52, 0.75		
	1 to < 3 years NBB use^d^, ^e^	151	2	1081	2	1232	0.64	0.54, 0.76		
	3 to < 5 years NBB use^d^, ^f^	138	1	898	2	1036	0.70	0.58, 0.84		
	≥ 5 years NBB use^d^, ^g^	142	1	1412	3	1554	0.45	0.38, 0.54		
Thyroid	No use	3713	91	18,672	91	22,385	Reference		0.710	0.037
	< 1 year NBB use^d^	81	2	286	1	367	1.41	1.09, 1.81		
	1 to < 3 years NBB use^d^, ^e^	72	2	327	2	399	1.09	0.84, 1.41		
	3 to < 5 years NBB use^d^, ^f^	62	2	302	1	364	1.01	0.77, 1.34		
	≥ 5 years NBB use^d^, ^g^	78	2	424	2	502	0.91	0.71, 1.17		
Pancreas	No use	4598	79	23,106	79	27,704	Reference		0.763	0.490
	< 1 year NBB use^d^	186	3	924	3	1110	0.96	0.82, 1.13		
	1 to < 3 years NBB use^d^, ^e^	226	4	1062	4	1288	1.03	0.89, 1.19		
	3 to < 5 years NBB use^d^, ^f^	201	3	1015	3	1216	0.97	0.83, 1.14		
	≥ 5 years NBB use^d^, ^g^	347	6	1662	6	2009	1.01	0.90, 1.15		
Kidney	No use	3463	85	17,265	85	20,728	Reference		0.342	0.725
	< 1 year NBB use^d^	106	3	516	3	622	0.98	0.79, 1.22		
	1 to < 3 years NBB use^d^, ^e^	123	3	558	3	681	1.05	0.86, 1.29		
	3 to < 5 years NBB use^d^, ^f^	92	2	513	3	605	0.87	0.69, 1.09		
	≥ 5 years NBB use^d^, ^g^	163	4	842	4	1005	0.92	0.77, 1.09		
Cervix	No use	1506	90	7393	88	8899	Reference		0.006	0.197
	< 1 year NBB use^d^	47	3	181	2	228	1.22	0.88, 1.69		
	1 to < 3 years NBB use^d^, ^e^	32	2	167	2	199	0.90	0.61, 1.32		
	3 to < 5 years NBB use^d^, ^f^	26	2	172	2	198	0.71	0.47, 1.09		
	≥ 5 years NBB use^d^, ^g^	36	2	260	3	296	0.65	0.45, 0.93		
Stomach	No use	2378	80	11,903	80	14,281	Reference		0.807	0.806
	< 1 year NBB use^d^	102	3	483	3	585	1.03	0.83, 1.29		
	1 to < 3 years NBB use^d^, ^e^	96	3	561	4	657	0.83	0.66, 1.04		
	3 to < 5 years NBB use^d^, ^f^	102	3	472	3	574	1.05	0.84, 1.31		
	≥ 5 years NBB use^d^, ^g^	172	6	826	6	998	1.02	0.86, 1.21		
Liver	No use	1473	78	7732	82	9205	Reference		0.075	0.435
	< 1 year NBB use^d^	77	4	265	3	342	1.40	1.07, 1.82		
	1 to < 3 years NBB use^d^, ^e^	69	4	314	3	383	1.12	0.85, 1.47		
	3 to < 5 years NBB use^d^, ^f^	54	3	259	3	313	1.06	0.78, 1.44		
	≥ 5 years NBB use^d^, ^g^	127	7	520	5	647	1.25	1.01, 1.54		
Brain	No use	2101	86	10,448	86	12,549	Reference		0.866	0.992
	< 1 year NBB use^d^	66	3	286	2	352	1.14	0.87, 1.50		
	1 to < 3 years NBB use^d^, ^e^	58	2	346	3	404	0.83	0.62, 1.11		
	3 to < 5 years NBB use^d^, ^f^	55	2	298	2	353	0.91	0.68, 1.22		
	≥ 5 years NBB use^d^, ^g^	92	4	421	3	513	1.08	0.85, 1.37		
Bladder	No use	2014	78	9973	77	11,987	Reference		0.242	0.962
	< 1 year NBB use^d^	75	3	495	4	570	0.72	0.56, 0.93		
	1 to < 3 years NBB use^d^, ^e^	111	4	538	4	649	0.99	0.80, 1.23		
	3 to < 5 years NBB use^d^, ^f^	95	4	473	4	568	0.97	0.77, 1.22		
	≥ 5 years NBB use^d^, ^g^	163	6	874	7	1037	0.90	0.75, 1.07		
Gallbladder	No use	1444	79	7210	79	8654	Reference		0.782	0.402
	< 1 year NBB use^d^	66	4	321	4	387	0.99	0.75, 1.30		
	1 to < 3 years NBB use^d^, ^e^	66	4	366	4	432	0.87	0.66, 1.14		
	3 to < 5 years NBB use^d^, ^f^	60	3	301	3	361	0.95	0.72, 1.27		
	≥ 5 years NBB use^d^, ^g^	106	6	505	6	611	1.02	0.82, 1.27		

Abbreviations: CI, confidence interval; NBB, nitrogen‐based bisphosphonates; OR, odds ratio; osteo, osteoporosis.

^a^ Excludes cases diagnosed prior to 1st July 2008.

^b^ Model adjusted for weighted Rx‐Risk score, and matched by age, SEIFA, remoteness and registered state.

^c^ *p* value of duration as a continuous variable using the median duration of each category. Results presented with and without no use of osteoporosis medicines used in the model.

^d^ No use of other osteoporosis medicines.

^e^ Minimum 1 year duration of use plus a minimum 292 defined daily doses.

^f^ Minimum 3 years duration of use plus a minimum 877 defined daily doses.

^g^ Minimum 5 years.

### 3.2 Breast Cancer

There were 90,992 eligible women diagnosed with breast cancer during the study period. Use of NBB was associated with around a 15% reduction in breast cancer risk (OR = 0.85, 95% CI: 0.83, 0.87) compared to no use of osteoporosis medicines. The reduction was slightly greater for 5 years or more of NBB use (OR = 0.83, 95% CI: 0.78, 0.88) compared to less than 1 year's use (OR = 0.90, 95% CI: 0.85, 0.97). Raloxifene was associated with a 34% reduced risk (OR = 0.66, 95% CI: 0.59, 0.73). Other osteoporosis medicines were used by fewer women than NBB, and while estimates indicated a reduced risk, the confidence intervals included the null (Table S6). The results for breast cancer were robust to sensitivity analyses where we adjusted for variables derived from prescription (diabetes, MHT use) (Table S9) and hospital (bilateral oophorectomy more than 6 months before index date, parity, osteoporosis/pathological fracture) records (Table S14).

### 3.3 Uterine Cancer

There were 15,362 eligible women diagnosed with uterine cancer during the study period. NBB use was associated with a 39% reduction in risk of uterine cancer (OR = 0.61, 95% CI: 0.57, 0.66) (Table 3) and this did not change substantively when we adjusted for diabetes medicine or MHT use (Table S9). The risk of uterine cancer was further reduced in women who had used NBB for 5 years or more (OR = 0.45, 95% CI: 0.38, 0.54) (Table 4). As for breast cancer, inverse associations were also observed for exclusive use of raloxifene (OR = 0.77, 95% CI: 0.61, 0.97) (Table 3), but also for use of non‐nitrogen‐based bisphosphonates (OR = 0.32, 95% CI: 0.13, 0.78) and strontium and/or denosumab (OR = 0.52, 95% CI: 0.36, 0.74) (Table S6).

When we restricted our analyses to women from Western Australia and excluded women who had a hysterectomy prior to index date from control selection, the association was only slightly weaker (OR = 0.66, 95% CI: 0.49, 0.88) compared to the unrestricted Western Australia data (OR = 0.63, 95% CI: 0.47, 0.84) (Table S14). When we additionally adjusted for hormone‐related covariates from health records (parity, bilateral oophorectomy), as well as any osteoporosis or likely osteoporotic fracture more than 6 months before index, the association weakened further (OR = 0.74, 95% CI: 0.55, 0.99).

Our quantitative bias analysis showed that if we had been able to adjust for obesity in the analysis, the inverse association between NBB use and uterine cancer may have attenuated slightly towards the null (Table S17). However, even when we used the upper 95% confidence interval for the association between obesity and endometrial cancer and used a low prevalence of obesity in bisphosphonate users of 50% of that of non‐users, the quantitative bias analysis still showed a 13% reduced risk of uterine cancer for NBB users.

### 3.4 Cervical Cancer

Overall, 2841 eligible women were diagnosed with cervical cancer during the study period. Use of NBB was associated with lower incidence of cervical cancer in women over 50 years compared to women who did not use osteoporosis medicines (OR = 0.83, 95% CI: 0.71, 0.97). The association was strongest among women with five or more years of NBB use (OR = 0.65, 95% CI: 0.45, 0.93). Adjusting for diabetes medicines and MHT in the sensitivity analysis using the concessional dataset did not change the estimates, although noting that the estimates were slightly weaker for this dataset (Table S9).

In sensitivity analyses using data from Western Australia, the inverse association between NBB use and cervical cancer attenuated slightly after excluding women with a hysterectomy (from OR = 0.82, 95% CI: 0.44, 1.54 to OR = 0.88, 95% CI: 0.47, 1.64) and also when we adjusted for a hospital‐recorded diagnosis of osteoporosis or likely osteoporotic fracture (OR = 0.94, 95% CI: 0.49, 1.80) notwithstanding the imprecision due to the relatively small number of eligible women diagnosed with cervical cancer in Western Australia during the study period.

### 3.5 Colorectal Cancer

There were 36,009 women diagnosed with colorectal cancer during the study period. While we did not observe any association between use of NBB and colorectal cancer (OR = 1.02, 95% CI: 0.99, 1.06), exclusive use of raloxifene was associated with a reduced risk of colorectal cancer (OR = 0.89, 95% CI: 0.79, 0.99) compared to no use of osteoporosis medicines.

### 3.6 Liver Cancer

There were 2998 women diagnosed with liver cancer during the study period. NBB use was associated with an increased risk of liver cancer compared to no use of osteoporosis medicines (OR = 1.15, 95% CI: 1.02, 1.29), which appeared to be mostly driven by short‐term use (< 1 year: OR = 1.40, 95% CI: 1.07, 1.82); however, use of more than 5 years was also associated with an increased risk (OR = 1.25, 95% CI: 1.01, 1.54) (Table 4).

### 3.7 Lung Cancer

There were 29,796 women diagnosed with lung cancer during the study period. Overall, NBB use was associated with an increase in lung cancer risk (OR = 1.16, 95% CI: 1.12, 1.21) but the magnitude of the association decreased with longer duration of NBB use (Table 4). Exclusive use of raloxifene was similarly associated with increased risk (OR = 1.18, 95% CI: 1.05, 1.33) compared to no use of osteoporosis medicines, but use of other osteoporosis medicines was not (non‐nitrogen‐based bisphosphonates: OR = 1.02, 95% CI: 0.72, 1.45, strontium and/or denosumab: OR = 0.92, 95% CI: 0.76, 1.11).

Quantitative bias analyses suggested that adjustment for smoking would only slightly attenuate the crude OR for lung cancer in NBB users (Table S18). Unpublished data from the QSkin study [37] indicated that smoking rates were not appreciably different between NBB users and non‐users in a comparable population; however, when we applied a high current smoker rate in NBB users (double the rate of non‐users), the crude OR attenuated to the null (standardised morbidity ratio: 0.97).

### 3.8 Results for Other Cancers

NBB use was associated with a reduced but not statistically significant risk of kidney cancer (OR = 0.93, 95% CI: 0.85, 1.02). Results from the duration of use analysis showed that the association was only present for three or more years of use in both the main analysis (Table 4) and sensitivity in the concessional cohort (Table S10). There was a suggestive association for use of NBBs and increased risk of thyroid cancer (OR = 1.11, 95% CI: 0.99, 1.24) (Table 3) that appeared to be restricted to short‐term (< 1‐year) use of NBB (OR = 1.41, 95% CI: 1.10, 1.80) (Table 4). Similarly, NBB use of less than 1 year was associated with a decreased risk of bladder cancer (OR = 0.72, 95% CI: 0.56, 0.93), but not for longer durations (Table 4). There were no significant associations between NBB use and melanoma, pancreatic, stomach, brain or gallbladder cancers.

## 4 Discussion

Our study found associations between NBB use and reductions in the risk of cancers which are linked to reproductive hormones. Specifically, we observed that NBB use compared to no osteoporosis medicines use was associated with a lower risk of breast, uterine and cervical cancers and that the reduction in risk was greatest for women who used NBB for more than 5 years. In contrast, NBBs were associated with an increased risk of lung and liver cancer. While relatively few women in our study used raloxifene or other osteoporosis medicines, we observed a lower risk of breast, colorectal, uterine and cervical cancer for women who used raloxifene and a reduced risk of uterine cancer for users of other osteoporosis medicines.

### 4.1 Breast and Gynaecological Cancers

Other studies have reported similar findings to ours for breast and gynaecological cancer. A recent meta‐analysis of observational studies [6] showed an inverse association between NBB use and breast (RR = 0.94, 95% CI: 0.90, 0.99 from 6 studies), endometrial (RR = 0.70, 95% CI: 0.54, 0.92 from five studies) and cervical (RR = 0.75, 95% CI: 0.55, 1.01 from 3 studies) cancers with some evidence of further reduction in risk with greater duration of use [6]. NBB (zoledronate) also appeared to reduce risk of breast cancer during 6 years of zoledronate treatment in an RCT of older women with osteopenia [4] and to reduce breast cancer recurrence in post‐menopausal women [38]. Antitumor effects of zoledronic acid on breast cancer have been shown to be more effective for post‐menopausal women due to the oestrogen deficient environments, independent of the oestrogen receptor status of the tumour [39]. A relationship is biologically plausible as, while bisphosphonates have a high affinity for bone and uptake by osteoclasts, they may also be endocytosed by tumour cells, tumour‐associated macrophages and other cells in the tumour environment [1]. In vitro studies have shown NBBs to have direct anti‐cancer effects, including disrupting the mevalonate pathway and reducing the viability of human endothelial cells [40]. They have also shown that bone bound NBBs can inhibit the growth of adjacent breast cancer cells [41].

There is existing evidence from clinical trials that use of raloxifene reduces the risk of breast cancer [42, 43, 44], in particular oestrogen receptor positive breast cancer. However, the evidence is not as clear for other cancers. Raloxifene is an oestrogen receptor antagonist and mice studies have shown raloxifene to be effective in preventing cervical cancer [45], however this has not been replicated in human studies. A randomised controlled trial investigating safety and adverse effects associated with raloxifene had only small numbers of endometrial (*n* = 14) and colorectal cancer (*n* = 50); therefore, they did not find significant associations (endometrial cancer: RR 0.9; 95% CI 0.3–2.7 [46], colorectal cancer: RR = 0.78, 95% CI 0.43, 1.43) [47] although the effect estimates were below one. A case–control study with 547 endometrial cancer cases found a significant reduced risk for use of raloxifene (OR = 0.50, 95% CI: 0.29, 0.85). Our large study provides further evidence that raloxifene may reduce other oestrogen‐receptor positive cancers, beyond breast cancer.

Confounding by indication is another possible explanation for our results. A history of osteoporosis or minimal trauma fracture may reflect long term low oestrogen exposure and subsequent lower risk of oestrogen‐responsive cancers [48] independent of bisphosphonate usage. This possibility is supported by the fact that users of other osteoporosis medicine (non‐nitrogen‐based bisphosphonates, strontium and/or denosumab) also had lower breast and uterine cancer incidence, noting that it is possible that these women also used bisphosphonates prior to these medicines. Several other factors argue against confounding by indication being the sole explanation for our results. Firstly, the effects of NBB use on risk of breast and uterine cancers were robust to sensitivity analyses for factors associated with oestrogen exposure (diabetes, MHT use, gynaecological surgery and parity). In addition, confounding by indication should not have affected the fracture prevention RCTs that examined breast cancer incidence [3, 4]; the one with the longer follow‐up showed preventive benefit although the other short‐term study did not. The few observational studies that restricted analyses to women with osteoporosis had relatively short follow‐up periods and very small case numbers limiting their power. Nevertheless, the effect estimates reported by two studies were similar to ours for breast [9, 49], and cervical [9] cancer, although the results for the third [6] were not. Several observational studies which adjusted for validated fracture risk scores reported a protective effect of bisphosphonates on breast [11, 15, 17] and uterine [14] cancer risk, and our emulated trial only including women indicated for NBB use found a protective effect for longer durations of use for ovarian cancer risk [50].

### 4.2 Other Cancers

Most other cancers were not clearly associated with NBB use. We did observe associations between NBB use and increased risk of lung, liver and thyroid cancer, although there were no clear patterns with increasing duration of use. Our quantitative bias analysis suggested that unmeasured confounding by smoking may account for the lung cancer association and there may also be unmeasured confounding due to chronic conditions associated with both osteoporosis and cancer incidence. Chronic liver disease [51] and chronic obstructive pulmonary disease [52] are both risk factors for osteoporosis, and for liver and lung cancer respectively, and our comorbidity score did not include medicines for these diseases. For thyroid cancer the increase in risk was highest with use commencing the year before diagnosis, which might be explained by increased ascertainment (overdiagnosis) due to greater contact with medical professionals [53]. Nevertheless, the associations with these cancers warrant further investigation.

### 4.3 Strengths and Limitations

Our study used objective, national data for prescriptions dispensed, cancer diagnoses and health insurance enrolment which removed the risk of recall and selection biases. Our inclusion of all eligible cases of the most frequently diagnosed solid cancers in Australian women allowed evaluation of the general effect of NBB on risk of cancers. The detailed prescription data enabled us to minimise the risk of misclassification of women who were exposed and not exposed to NBB and calculate total dose and duration of use. While we cannot know whether the medicines were consumed as prescribed, we required two dispensings within 12 months to ascertain exposure, therefore we were unlikely to misclassify non‐users as users. Misclassification of this nature would most likely have biased associations towards the null. The main limitation of our study was that we did not have comprehensive data on potential confounders for the whole population. It is possible that those who have better adherence to bisphosphonates are more likely to have other healthy behaviours that reduce cancer risk. However, we used state‐based hospital and health records in sensitivity analyses and quantitative bias analyses to explore the potential for confounding for our results. We also made multiple comparisons in our analyses therefore it is possible that some of the statistically significant associations we observed occurred due to chance. However, we performed the nested case–control analysis for each cancer in isolation, and the trends with increasing duration of exposure suggest this is a less likely explanation for our results.

## 5 Conclusion

Our large study investigating the association between NBB use and different types of cancer for women aged over 50 years found associations between NBB use and a reduction in breast, uterine and cervical cancers that were stronger for longer durations of use. Further research is needed to investigate these associations by histological subtype. We found no association for most other cancer types; however, our exploratory study identified associations between some medicine types and certain cancers that may provide new insights for cancer aetiology although they require replication by other studies.

## Author Contributions

**Karen M. Tuesley:** writing – original draft, formal analysis, investigation, methodology, conceptualization. **Fiona Caristo:** writing – original draft, formal analysis, investigation, methodology, visualization. **Katrina Spilsbury:** writing – review and editing, funding acquisition, formal analysis, methodology, conceptualization. **Sallie‐Anne Pearson:** writing – review and editing, funding acquisition, conceptualization. **Peter Donovan:** writing – review and editing, funding acquisition, conceptualization. **Michael D. Coory:** writing – review and editing, funding acquisition, conceptualization. **Christopher B. Steer:** writing – review and editing, funding acquisition, conceptualization. **Susan J. Jordan:** writing – review and editing, conceptualization, methodology, supervision, project administration, funding acquisition, data curation, investigation, validation.

## Funding

This work was supported by the Australian National Health and Medical Research Council (grant number GNT1121151).

## Ethics Statement

The study was approved by the following Human Research Ethics Committees (HRECs): The University of Queensland (2018001882), the QIMR Berghofer Medical Research Institute (P2214), the Australian Institute of Health and Welfare (EO2016/3/276), Australian Capital Territory Health on 3 April 2017, New South Wales Population and Health Services, Western Australia Department of Health, Queensland Department of Health, Tasmania Health and Medical and Northern Territory Department of Health.

## Conflicts of Interest

C.B.S. is a medical oncologist and in this role has received honoraria for advisory board membership from Bayer, Bristol Myers Squibb (BMS), Janssen and Merck Sharp and Dohme (MSD) and speakers' fees from Astra Zeneca, Eisai, Bristol Myers Squibb and Merck Sharp and Dohme. None of these were related to the research question in this study. The other authors declare no conflicts of interest.

## Supporting information


**Figure S1:** Periods covered by the datasets.
**Table S1:** Cancer site ICD‐O codes.
**Table S2:** PBS codes for bisphosphonate medication.
**Table S3:** Rx risk comorbidity categories, weights and ATC codes included in the RX risk comorbidity index.
**Table S4:** Diagnosis codes for osteoporosis and pathological fracture.
**Table S5:** Hospital procedure codes for hysterectomy for Western Australian women.
**Table S6:** Association between other osteoporosis medication use with cancer incidence in the national cohort.
**Table S7:** Characteristics of women diagnosed with cancer, by matching variables, concessional data.
**Table S8:** Characteristics of women diagnosed with cancer, medication use from PBS, concessional data.
**Table S9:** Association between osteoporosis medication use and cancers, adjusting for diabetes medicines and menopausal hormone therapy in the concessional dataset.
**Table S10:** Association between duration of exclusive NBB use and cancers, concessional dataset.
**Table S11:** Characteristics of women diagnosed with cancer in Western Australian.
**Table S12:** Characteristics of women diagnosed with cancer, medication use from PBS, WA data.
**Table S13:** Characteristics of women diagnosed with cancer, medication use from PBS, WA data (controls have no prior hysterectomy).
**Table S14:** Association between nitrogen‐based bisphosphonates and raloxifene and cancers, WA data, adjusted for Rx‐Risk scores and covariates from health records.
**Table S15:** Adjusted (margins at mean age) proportion of bisphosphonate users and non‐users in each body mass index category from unpublished QSkin study data.
**Table S16:** Proportion of bisphosphonate users and non‐users with reported smoking status (current, former, never) from unpublished QSkin study data.
**Table S17:** Quantitative bias analysis for the effect of obesity on the association between bisphosphonate use and uterine cancer.
**Table S18:** Quantitative bias analysis for the effect of smoking status on the association between bisphosphonate use and lung cancer.

## Data Availability

The data that support the findings of this study are available from the authors upon reasonable request and with permission from the Australian Institute of Health and Welfare and data custodians.
