# Supplementary material for: Nitrogen‐Based Bisphosphonate Use and Risk of Cancer in Women Aged 50 Years and Older: A National Data Linkage Study
Source: Int J Cancer. 2026 May 20;159(7):1669–85. doi: 10.1002/ijc.70535 (PMC13432198; doi:10.1002/ijc.70535)
Supplement: Supplementary file 1 — Figure S1: Periods covered by the datasets. Table S1: Cancer site ICD‐O codes. Table S2: PBS codes for bisphosphonate medication. Table S3: Rx risk comorbidity categories, weights and ATC codes included in the RX risk comorbidity index. Table S4: Diagnosis codes for osteoporosis and pathological fracture. Table S5: Hospital procedure codes for hysterectomy for Western Australian women. Table S6: Association between other osteoporosis medication use with cancer incidence in the national cohort. Table S7: Characteristics of women diagnosed with cancer, by matching variables, concessional data. Table S8: Characteristics of women diagnosed with cancer, medication use from PBS, concessional data. Table S9: Association between osteoporosis medication use and cancers, adjusting for diabetes medicines and menopausal hormone therapy in the concessional dataset. Table S10: Association between duration of exclusive NBB use and cancers, concessional dataset. Table S11: Characteristics of women diagnosed with cancer in Western Australian. Table S12: Characteristics of women diagnosed with cancer, medication use from PBS, WA data. Table S13: Characteristics of women diagnosed with cancer, medication use from PBS, WA data (controls have no prior hysterectomy). Table S14: Association between nitrogen‐based bisphosphonates and raloxifene and cancers, WA data, adjusted for Rx‐Risk scores and covariates from health records. Table S15: Adjusted (margins at mean age) proportion of bisphosphonate users and non‐users in each body mass index category from unpublished QSkin study data. Table S16: Proportion of bisphosphonate users and non‐users with reported smoking status (current, former, never) from unpublished QSkin study data. Table S17: Quantitative bias analysis for the effect of obesity on the association between bisphosphonate use and uterine cancer. Table S18: Quantitative bias analysis for the effect of smoking status on the association between bisphosphonate use and lung cancer. [file IJC-159-1669-s001.pdf]

Supplementary Materials for:

Nitrogen-based bisphosphonate use and risk of cancer in women aged 50 years and older: A national data linkage study.

Karen M. Tuesley, Fiona Caristo, Katrina Spilsbury, Sallie-Anne Pearson, Peter Donovan, Michael D. Coory, Christopher B. Steer, Susan J. Jordan.

Table of Contents

Table of Contents 1
Supplementary Methods 3
  Concessional eligibility criteria for the Pharmaceutical Benefits Scheme (PBS) 3
  References 5
Supplementary Figures 6
Supplementary Tables 7

List of Supplementary Figures

Supplementary Figure 1: Periods covered by the datasets 6

List of Supplementary Tables

Supplementary Table 1: Cancer site ICD-O codes 7
Supplementary Table 2: PBS codes for bisphosphonate medication 8
Supplementary Table 3: Rx Risk Comorbidity categories, weights and ATC Codes included in the RX Risk comorbidity index 9
Supplementary Table 4: Diagnosis codes for osteoporosis and pathological fracture 10
Supplementary Table 5: Hospital procedure codes for hysterectomy for Western Australian women 10
Supplementary Table 6: Association between other osteoporosis medication use with cancer incidence in the national cohort. 11

Supplementary Table 7: Characteristics of women diagnosed with cancer, by matching variables, Concessional data 13
Supplementary Table 8: Characteristics of women diagnosed with cancer, medication use from PBS, Concessional data 14
Supplementary Table 9: Association between osteoporosis medication use and cancers, adjusting for diabetes medicines and menopausal hormone therapy in the concessional dataset 15
Supplementary Table 10: Association between duration of exclusive NBB use and cancers, concessional dataset 17
Supplementary Table 11: Characteristics of women diagnosed with cancer in Western Australian 19
Supplementary Table 12: Characteristics of women diagnosed with cancer, medication use from PBS, WA data 20
Supplementary Table 13: Characteristics of women diagnosed with cancer, medication use from PBS, WA data (controls have no prior hysterectomy) 21
Supplementary Table 14: Association between nitrogen-based bisphosphonates and raloxifene and cancers, WA data, adjusted for Rx-Risk scores and covariates from health records 22
Supplementary Table 15: Adjusted (margins at mean age) proportion of bisphosphonate users and non-users in each body mass index category from unpublished QSkin study data 23
Supplementary Table 16: Proportion of bisphosphonate users and non-users with reported smoking status (current, former, never) from unpublished QSkin study data 23
Supplementary Table 17: Quantitative bias analysis for the effect of obesity on the association between bisphosphonate use and uterine cancer 24
Supplementary Table 18: Quantitative bias analysis for the effect of smoking status on the association between bisphosphonate use and lung cancer 25

Supplementary Methods

The following supplementary methods have been adapted from the supplementary materials from our previous published study.[1]

Concessional eligibility criteria for the Pharmaceutical Benefits Scheme (PBS)

In Australia, the amount an individual pays (the co-payment) for a prescription medicine listed on the PBS depends on their beneficiary status, defined as either general or concessional. Up until 2012, PBS medicines dispensed to general beneficiaries that cost less than the general beneficiary co-payment were not recorded in the PBS database (as the individual paid the entire cost with no government subsidy). All bisphosphonates included in our study were above co-payment prior to 2012 and therefore captured in the PBS records for all women in our study. However, cheaper medicines, such as certain MHT medicines, may not be recorded for women who were general beneficiaries prior to 2012. We therefore identified a subgroup of women who were concessional beneficiaries, for whom all PBS medicines were recorded, and we conducted sensitivity analysis to adjust for MHT use. A woman was defined as a concessional beneficiary in a given calendar year when she filled at least one prescription with a concessional status during that year. Women were eligible for selection into the concessional beneficiary subgroup once they had two years with concessional beneficiary status and were excluded from selection if they were no longer concessional (only general beneficiary prescriptions dispensed during a calendar year).

Methods for quantitative bias analysis (QBA)

We used QBA[2,3] to quantify the potential effect of unmeasured confounders, focussing on two specific relationships. Firstly, we assessed the effects of adjusting for obesity on our analyses of the association between NBB and uterine cancer; secondly, we assessed the effects of adjusting for smoking on our analyses of the association between NBB and lung cancer.

To ascertain the prevalence of BMI groups and smoking status amongst users and non-users of bisphosphonates, we used data from over 23,000 Queensland women who participated in the

QSkin study from 2010 to 2014 aged over 45 years.[4] We used the prevalence in users and non-users for obesity groups (BMI: 30-<35, 35-<40 and 40+) and smoking status (current, past, never).

We used estimates of the association between obesity and risk of endometrial cancer from a pooled analysis from the Epidemiology of Endometrial Cancer Consortium (E2C2).[5] Type I and II tumours were separately analysed, with differing estimates, therefore we first used Type I estimates in our QBA, and then used Type II estimates. We used the pooled odds ratio (OR) for the 30-34.9, 35-39.9 and 40+ BMI groups (BMI of <25 was the reference group) and the upper 95% confidence interval (CI) value to explore the potential bias. We applied the QBA methods for polytomous confounders,[2] first using BMI of 40+ as the highest-level confounder and BMI of 35-<40 as the mid-level confounder. We repeated the analysis combining 35-<40 and 40+ BMI as the highest-level confounder, and the 30-<35 BMI group as the mid-level confounder. We additionally performed a sensitivity analysis where the prevalence of obesity in the bisphosphonate user group was half of that in the non-user group.

We used estimates of the association between smoking (current and former) and lung cancer from a meta-analysis of tobacco smoking and cancer.[6] In our QBA model, we used the estimate for current smoker as the highest-level confounder, and former smokers as the mid-level confounder. We repeated the analyses using the upper 95% confidence interval of the association between smoking and lung cancer. We also performed a sensitivity analysis with current smoking in the bisphosphonate user group twice that in the non-user group.

We used the standardised morbidity ratio to estimate the risk ratio for the association between nitrogen-based bisphosphonate use and risk of EOC.

References

1. Tuesley KM, Webb PM, Protani MM, Spilsbury K, Pearson SA, Coory MD, et al. Nitrogen-based Bisphosphonate Use and Ovarian Cancer Risk in Women Aged 50 Years and Older. J Natl Cancer Inst. 2022;114(6):878-84.
2. Lash TL, Fox MP, MacLehose RF, Maldonado G, McCandless LC, Greenland S. Good practices for quantitative bias analysis. Int J Epidemiol. 2014;43(6):1969-85.
3. Lash TL, Fox MP, Fink AK. Applying Quantitative Bias Analysis to Epidemiologic Data. 1st ed. 2009. ed. New York, NY: Springer New York : Imprint: Springer; 2009.
4. Olsen CM, Green AC, Neale RE, Webb PM, Cicero RA, Jackman LM, et al. Cohort profile: the QSkin Sun and Health Study. Int J Epidemiol. 2012;41(4):929-i.
5. Setiawan VW, Yang HP, Pike MC, McCann SE, Yu H, Xiang YB, et al. Type I and II endometrial cancers: have they different risk factors? J Clin Oncol. 2013;31(20):2607-18.
6. Gandini S, Botteri E, Iodice S, Boniol M, Lowenfels AB, Maisonneuve P, et al. Tobacco smoking and cancer: a meta-analysis. Int J Cancer. 2008;122(1):155-64.

Supplementary Figures

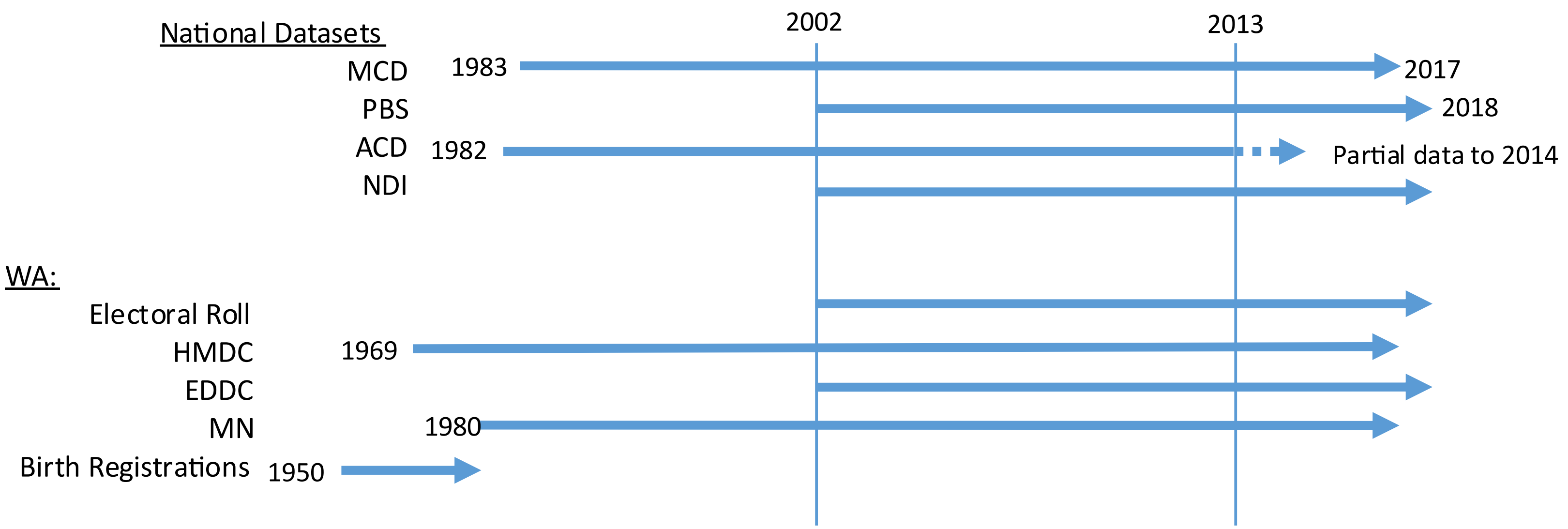

Supplementary Figure 1: Periods covered by the datasets

ACD: Australian Cancer Database; EDDC: Emergency Department Data Collection; HMDC: Hospital Morbidity Data Collection; MCD: Medicare Consumer Database; MN: Midwives Notifications; NDI: National Death Index; PBS: Pharmaceutical Benefits Scheme; WA: Western Australia.

Supplementary Tables

Supplementary Table 1: Cancer site ICD-O codes

Cancer site	ICD-O codes[a]
Breast	C50
Colorectal	C18, C19, C20, C21
Melanoma of the skin	C44 (C43 in ICD-10)
Lung	C33, C34
Uterus	C54, C55
Thyroid	C73
Pancreas	C25
Kidney	C64
Cervix	C53
Stomach	C16
Liver	C22
Brain	C71
Bladder	C67
Gallbladder and extrahepatic ducts	C23, C24

[a] Invasive cancers only. In-situ and borderline tumours were not included.

Supplementary Table 2: PBS codes for bisphosphonate medication

Medication Type	PBS Item Code	DDD (days)
**Nitrogen-based bisphosphonates**		
Alendronate	02194L	7
Alendronate	02215N	7
Alendronate	02224C	7
Alendronate	02273P	28
Alendronate	08090T[a]	4
Alendronate	08102K[a]	1
Alendronate	08511Y[a]	7
Alendronate	09012H[a]	7
Alendronate	09183H[a]	7
Alendronate	09351E[a]	28
Risedronate	02191H	7
Risedronate	02220W	28
Risedronate	02254P[a]	28
Risedronate	04059P[a]	28
Risedronate	04380M[a]	28
Risedronate	04443W[a]	1
Risedronate	04444X[a]	7
Risedronate	08481J[a]	1
Risedronate	08621R[a]	7
Risedronate	08899J[a]	28
Risedronate	08972F[a]	7
Risedronate	08973G[a]	28
Risedronate	08974H[a]	28
Risedronate	09147K[a]	28
Risedronate	09391G[a]	30
Risedronate	08482K[a]	6
Zoledronic Acid	06371H[a]	1 course
Zoledronic Acid	09288W[a]	1 course
Zoledronic Acid	09350D[a]	1 course
Zoledronic Acid	09653C[a]	1 course
Zoledronic Acid	10542W	1 course
Zoledronic Acid	10548E	1 course
Zoledronic Acid	10554L	1 course
Zoledronic Acid	10555M	1 course
Zoledronic Acid	10561W	1 course
Zoledronic Acid	10571J	1 course
**Raloxifene**		
Raloxifene	08363E[a]	1
**Other osteoporosis medications**		
Etidronate	02920Q[a]	0.5
Etidronate	08056B[a]	14
Strontium	03036T[a]	1
Denosumab	05457F[a]	1 course

DDD: defined daily dose; PBS: Pharmaceutical Benefits Scheme.
[a] Included in the nested case-control analysis.

Supplementary Table 3: Rx Risk Comorbidity categories, weights and ATC Codes included in the RX Risk comorbidity index

Rx Risk Comorbidity Category[a]	Weight for Rx Risk score	ATC Codes
Anticoagulants	1	B01AA03-B01AB06, B01AE07, B01AF01, B01AF02, B01AX05
Anitplatelets	2	B01AC04–B01AC30
Arrhythmia	2	C01AA05, C01BA01–C01BD01, C07AA07
Congestive heart failure	2	C03DA02–C03DA99, C07AB02 (if PBS item code is 8732N, 8733P, 8734Q, 8735R, 08818D), C07AB07, C07AG02, C07AB12, and both of (C03CA01–C03CC01) and (C09AA01–C09AX99, C09CA01–C09CX99)
Diabetes	2	A10AA01–A10BX99
Gastrooesophageal reflux disease	0	A02BA01–A02BX05
Hyperlipidaemia	-1	A10BH03, C10AA01–C10BX09
Hypertension	-1	C03AA01–C03BA11, C03DB01-C03DB99, C03EA01, C09BA02–C09BA09, C09DA02–C09DA08, C02AB01–C02AC05, C02DB02–C02DB99, (C03CA01–C03CCO1) or (C09CA01–C09CX99) but not both groups.
Hyperthyroidism	2	H03BA02, H03BB01
Hypothyroidism	0	H03AA01–H03AA02
Ischaemic heart disease: angina	2	C01DA02–C01DA14, C01DX16, C08EX02
Ischaemic heart disease: hypertension	-1	C07AA01–C07AA06, C07AA08–C07AB03, C07AB02 (if PBS item code is not 8732N, 8733P, 8734Q, 8735R, 08818D) C07AG01, C08CA01–C08DB01, C09DB01–C09DB04, C09DX01, C09BB02–C09BB10, C09DX03, C10BX03
Inflammation/pain	-1	M01AB01–M01AH06
Malignancies	2	L01AA01–L01XX41
Osteoporosis/Paget's	-1	M05BA01–M05BB05, M05BX03, M05BX04, G03XC01, H05AA02
Pain	3	N02AA01–N02AX02, N02AX06, N02AX52, N02BE51
Pulmonary hypertension	6	C02KX01–C02KX05, C02KX
Steroid-responsive disease	2	H02AB01–H02AB10
Transplant	0	L04AA01- L04AA21, L04AD01-L04AD02

[a] Comorbidity categories with a Rx Risk weighting excluded from our calculation due to PBS item codes not being available in our data set: Alcohol dependency, Allergies, Anxiety, Bipolar disorder, Dementia, Gout, Hyperkalaemia, Liver failure, Migraine, Parkinson's disease, Psychotic Illness, Renal disease, Smoking cessation. For each of these, less than 12% of the Department of Veteran Affairs cohort were identified as having the condition and unlikely to affect the weighted RX risk score.

Supplementary Table 4: Diagnosis codes for osteoporosis and pathological fracture

Diagnosis	ACHI	ICD-9-CM	ICD-8
Osteoporosis	M80 M81 [a]	7330	7230
Fractures most likely due to osteoporosis[34]	M80 S720 S220 S221 S320 S327 S321 S525 S322 S323 S324 S325 S328	7331 820 8052 8053 8062 8063 8054 8055 8064 8065 8134 8138 808	7232

ACHI: Australian Classification of Health Interventions; ICD: International Classification of Diseases.
[a] We excluded M82 as this includes osteoporosis resulting from another underlying disease process, such as multiple myelomatosis or endocrine disorders.

Supplementary Table 5: Hospital procedure codes for hysterectomy for Western Australian women

Procedure	ACHI	ICD-9-CM	ICPM	COSO
Hysterectomy	3565300-3565304, 3565700, 3565800, 3566100, 3566400, 3566401, 3566700, 3566701, 3567000, 3567300-3567302, 3575000, 3575300-3575302, 3575600-3575603, 9044300, 9044800-9044802	683-689	5682-5687	0690, A690, B690, C690, 0691, 0692, A692, 0693, A693, B693, C693, 0694, 0696, 0744, 0764

ACHI: Australian Classification of Health Interventions, COSO: Code of Surgical Operations, Commonwealth Department of Health, Canberra 1968; ICD: International Classification of Diseases; ICPM: International Classification of Procedures in Medicine, WHO Geneva 1978

Supplementary Table 6: Association between other osteoporosis medication use with cancer incidence in the national cohort.

Cancer site	Osteoporosis medication	Cases		Controls		Adjusted Model[a]	
		n	%	n	%	OR	95% CI
Breast	Non-user	82,550	91	405,786	89		Reference
	nNBB only	73	0	403	0	0.87	0.68,1.12
	Strontium &/or denosumab only	272	0	1,487	0	0.88	0.77,1.00
	NBB and other osteo medication/s	734	1	4,759	1	0.74	0.68,0.80
Colorectal	Non-user	29,727	83	148,917	83		Reference
	nNBB only	59	0	272	0	1.08	0.82,1.44
	Strontium &/or denosumab only	159	0	816	0	0.97	0.82,1.15
	NBB and other osteo medication/s	606	2	3,163	2	0.95	0.87,1.04
Melanoma	Non-user	23,546	87	117,782	87		Reference
	nNBB only	23	0	146	0	0.79	0.51,1.22
	Strontium &/or denosumab only	94	0	497	0	0.94	0.75,1.18
	NBB and other osteo medication/s	324	1	1,733	1	0.93	0.83,1.05
Lung	Non-user	24,277	81	125,679	84		Reference
	nNBB only	39	0	181	0	1.02	0.72,1.45
	Strontium &/or denosumab only	131	0	704	0	0.92	0.76,1.11
	NBB and other osteo medication/s	595	2	2,422	2	1.19	1.08,1.30
Uterus	Non-user	14,261	93	68,446	89		Reference
	nNBB only	<6 [b]		74	0	0.32	0.13,0.78
	Strontium &/or denosumab only	<35 [b]		303	0	0.52	0.36,0.74
	NBB and other osteo medication/s	102	1	832	1	0.57	0.46,0.70
Thyroid	Non-user	5,642	91	28,392	92		Reference
	nNBB only	<6 [b]		18	0	1.12	0.37,3.38
	Strontium &/or denosumab only	11	0	84	0	0.66	0.35,1.24
	NBB and other osteo medication/s	58	1	278	1	1.03	0.77,1.37
Pancreas	Non-user	7,658	81	38,492	81		Reference
	nNBB only	<15 [b]		93	0	0.72	0.41,1.27
	Strontium &/or denosumab only	52	1	277	1	0.91	0.67,1.22
	NBB and other osteo medication/s	193	2	903	2	1.02	0.87,1.20
Kidney	Non-user	5,589	86	27,832	86		Reference
	nNBB only	<6 [b]		38	0	0.50	0.18,1.39
	Strontium &/or denosumab only	20	0	133	0	0.72	0.45,1.15
	NBB and other osteo medication/s	97	1	478	1	0.97	0.77,1.21
Cervix	Non-user	2,577	91	12,648	89		Reference
	nNBB only	<6 [b]		19	0	0.25	0.03,1.87
	Strontium &/or denosumab only	<10 [b]		46	0	0.63	0.27,1.47
	NBB and other osteo medication/s	27	1	153	1	0.83	0.55,1.25
Stomach	Non-user	4,195	81	21,189	82		Reference
	nNBB only	<10 [b]		34	0	1.03	0.45,2.32
	Strontium &/or denosumab only	23	0	110	0	1.02	0.65,1.61
	NBB and other osteo medication/s	100	2	424	2	1.16	0.92,1.44
Liver	Non-user	2,402	80	12,407	83		Reference
	nNBB only	<6 [b]		23	0	0.86	0.29,2.49
	Strontium &/or denosumab only	23	1	74	0	1.55	0.97,2.49
	NBB and other osteo medication/s	51	2	248	2	1.03	0.76,1.40
Brain	Non-user	3,433	87	17,152	87		Reference
	nNBB only	<6 [b]		27	0	0.19	0.03,1.37
	Strontium &/or denosumab only	<15 [b]		70	0	0.93	0.51,1.68
	NBB and other osteo medication/s	57	1	289	1	0.99	0.74,1.33

Bladder	Non-user	3,455	79	17,304	79		Reference
	nNBB only	<15 [b]		40	0	1.19	0.59,2.40
	Strontium &/or denosumab only	17	0	104	0	0.80	0.48,1.33
	NBB and other osteo medication/s	101	2	391	2	1.25	1.00,1.56
Gallbladder	Non-user	2,407	81	12,004	81		Reference
	nNBB only	<6[b]		23	0	0.84	0.29,2.43
	Strontium &/or denosumab only	16	1	83	1	0.94	0.55,1.62
	NBB and other osteo medication/s	47	2	289	2	0.78	0.57,1.07

NBB: nitrogen-based bisphosphonates; nNBB: non-nitrogen-based bisphosphonates; OR: odds ratio; CI: confidence interval

a Models adjusted for weighted Rx-Risk score, and matched by age, SEIFA, remoteness and registered state.

b Cell numbers have been suppressed for privacy reasons.

Supplementary Table 7: Characteristics of women diagnosed with cancer, by matching variables, Concessional data

	Breast	Colorectal	Melanoma	Lung	Uterus	Thyroid	Pancreas	Kidney	Cervix	Stomach	Liver	Brain	Bladder	Gallbladder
Total Cases	N=51,343	N=27,770	N=16,494	N=23,495	N=9,341	N=3,064	N=7,641	N=4,685	N=1,733	N=4,113	N=2,434	N=2,613	N=3,731	N=2,387
Index age (y), mean (SD)	71.7 (10.2)	76.7 (9.3)	74.1 (10.2)	74.2 (9.4)	71.1 (9.7)	68.4 (10.1)	77.6 (9.5)	73.6 (10.0)	71.2 (11.5)	77.1 (9.8)	75.7 (10.3)	73.9 (9.4)	79.1 (9.3)	77.5 (9.3)
Index age (y)														
50-59y	6,623 (13%)	1,333 (5%)	1,529 (9%)	1,772 (8%)	1,229 (13%)	710 (23%)	293 (4%)	476 (10%)	361 (21%)	226 (5%)	220 (9%)	203 (8%)	110 (3%)	96 (4%)
60-69y	17,294 (34%)	5,310 (19%)	4,348 (26%)	6,135 (26%)	3,254 (35%)	1,037 (34%)	1,428 (19%)	1,208 (26%)	435 (25%)	756 (18%)	443 (18%)	680 (26%)	563 (15%)	413 (17%)
70-79y	15,716 (31%)	10,155 (37%)	5,564 (34%)	8,727 (37%)	2,986 (32%)	888 (29%)	2,570 (34%)	1,672 (36%)	490 (28%)	1,394 (34%)	838 (34%)	999 (38%)	1,166 (31%)	875 (37%)
80-89y	9,681 (19%)	9,221 (33%)	4,136 (25%)	5,974 (25%)	1,625 (17%)	378 (12%)	2,683 (35%)	1,130 (24%)	372 (21%)	1,391 (34%)	785 (32%)	639 (24%)	1,454 (39%)	819 (34%)
>=90y	2,029 (4%)	1,751 (6%)	917 (6%)	887 (4%)	247 (3%)	51 (2%)	667 (9%)	199 (4%)	75 (4%)	346 (8%)	148 (6%)	92 (4%)	438 (12%)	184 (8%)
State of residence at Medicare enrolment														
NSW	16,962 (33%)	9,345 (34%)	5,849 (35%)	8,072 (34%)	2,998 (32%)	1,264 (41%)	2,767 (36%)	1,605 (34%)	637 (37%)	1,372 (33%)	937 (38%)	845 (32%)	1,292 (35%)	726 (30%)
ACT	623 (1%)	326 (1%)	165 (1%)	218 (1%)	106 (1%)	26 (1%)	72 (1%)	55 (1%)	15 (1%)	31 (1%)	26 (1%)	25 (1%)	26 (1%)	22 (1%)
VIC	12,867 (25%)	6,965 (25%)	3,427 (21%)	5,834 (25%)	2,515 (27%)	659 (22%)	1,955 (26%)	1,158 (25%)	394 (23%)	1,193 (29%)	656 (27%)	762 (29%)	881 (24%)	686 (29%)
QLD	10,044 (20%)	5,489 (20%)	3,970 (24%)	4,290 (18%)	1,832 (20%)	605 (20%)	1,268 (17%)	887 (19%)	362 (21%)	659 (16%)	372 (15%)	411 (16%)	708 (19%)	421 (18%)
SA/NT	4,814 (9%)	2,533 (9%)	1,228 (7%)	2,056 (9%)	924 (10%)	158 (5%)	663 (9%)	438 (9%)	125 (7%)	392 (10%)	200 (8%)	231 (9%)	374 (10%)	236 (10%)
WA	4,591 (9%)	2,237 (8%)	1,390 (8%)	2,243 (10%)	724 (8%)	283 (9%)	691 (9%)	394 (8%)	138 (8%)	342 (8%)	187 (8%)	258 (10%)	313 (8%)	226 (9%)
TAS	1,442 (3%)	875 (3%)	465 (3%)	782 (3%)	242 (3%)	69 (2%)	225 (3%)	148 (3%)	62 (4%)	124 (3%)	56 (2%)	81 (3%)	137 (4%)	70 (3%)
SEIFA														
1 (most disadvantaged)	11,393 (22%)	6,089 (22%)	3,406 (21%)	6,178 (26%)	2,290 (25%)	806 (26%)	1,780 (23%)	1,151 (25%)	517 (30%)	1,022 (25%)	671 (28%)	561 (21%)	852 (23%)	559 (23%)
2	11,529 (22%)	6,281 (23%)	4,056 (25%)	5,477 (23%)	2,056 (22%)	663 (22%)	1,648 (22%)	1,070 (23%)	417 (24%)	866 (21%)	491 (20%)	597 (23%)	887 (24%)	546 (23%)
3	10,479 (20%)	5,684 (20%)	3,411 (21%)	4,813 (20%)	1,876 (20%)	584 (19%)	1,568 (21%)	964 (21%)	325 (19%)	808 (20%)	472 (19%)	529 (20%)	699 (19%)	461 (19%)
4	9,583 (19%)	5,152 (19%)	3,015 (18%)	3,874 (16%)	1,746 (19%)	530 (17%)	1,452 (19%)	819 (17%)	265 (15%)	788 (19%)	433 (18%)	487 (19%)	682 (18%)	463 (19%)
5 (least disadvantaged)	8,359 (16%)	4,564 (16%)	2,606 (16%)	3,153 (13%)	1,373 (15%)	481 (16%)	1,193 (16%)	681 (15%)	209 (12%)	629 (15%)	367 (15%)	439 (17%)	611 (16%)	358 (15%)
Remoteness														
Major City	33,967 (66%)	17,872 (64%)	10,052 (61%)	15,445 (66%)	6,160 (66%)	2,171 (71%)	5,137 (67%)	3,051 (65%)	1,159 (67%)	2,924 (71%)	1,817 (75%)	1,754 (67%)	2,514 (67%)	1,618 (68%)
Inner Regional	12,181 (24%)	6,845 (25%)	4,501 (27%)	5,522 (24%)	2,147 (23%)	597 (19%)	1,721 (23%)	1,151 (25%)	384 (22%)	828 (20%)	404 (17%)	597 (23%)	845 (23%)	521 (22%)
Outer Regional	4,654 (9%)	2,731 (10%)	1,764 (11%)	2,239 (10%)	885 (9%)	254 (8%)	707 (9%)	431 (9%)	163 (9%)	330 (8%)	184 (8%)	232 (9%)	338 (9%)	214 (9%)
Remote & Very Remote	541 (1%)	322 (1%)	177 (1%)	289 (1%)	149 (2%)	42 (1%)	76 (1%)	52 (1%)	27 (2%)	31 (1%)	29 (1%)	30 (1%)	34 (1%)	34 (1%)

SD: standard deviation; SEIFA: Socio-Economic Indexes for Areas; y: years.

Supplementary Table 8: Characteristics of women diagnosed with cancer, medication use from PBS, Concessional data

	Breast Cancer		Colorectal Cancer		Melanoma		Lung Cancer		Uterine Cancer	
**Category**	Case	Control	Case	Control	Case	Control	Case	Control	Case	Control
Total	N=51,343	N=256,640	N=27,770	N=138,803	N=16,494	N=82,445	N=23,495	N=117,452	N=9,341	N=46,694
Osteoporosis medications (a)										
Non-user	44,163 (86%)	214,636 (84%)	21,970 (79%)	109,480 (79%)	13,440 (81%)	66,911 (81%)	18,352 (78%)	95,458 (81%)	8,388 (90%)	39,262 (84%)
NBB only	5,888 (11%)	33,344 (13%)	4,685 (17%)	23,291 (17%)	2,471 (15%)	12,245 (15%)	4,083 (17%)	17,502 (15%)	753 (8%)	5,943 (13%)
Raloxifene only	340 (1%)	2,604 (1%)	335 (1%)	1,851 (1%)	185 (1%)	976 (1%)	322 (1%)	1,420 (1%)	75 (1%)	473 (1%)
nNBB only	59 (0%)	335 (0%)	57 (0%)	280 (0%)	20 (0%)	160 (0%)	33 (0%)	171 (0%)	<6	80 (0%)
Strontium &/or denosumab only	224 (0%)	1,198 (0%)	137 (0%)	838 (1%)	83 (1%)	420 (1%)	122 (1%)	622 (1%)	<35	207 (0%)
NBB and other osteo medication/s	649 (1%)	4,374 (2%)	571 (2%)	2,948 (2%)	287 (2%)	1,690 (2%)	560 (2%)	2,198 (2%)	90 (1%)	700 (1%)
>1 osteo medication, excluding NBB	20 (0%)	149 (0%)	15 (0%)	115 (0%)	8 (0%)	43 (0%)	23 (0%)	81 (0%)	0 (0%)	29 (0%)
Weighted RxRisk V Score (mean(SD))	1.1 (2.8)	1.1 (2.7)	1.4 (2.9)	1.3 (2.9)	1.2 (2.9)	1.2 (2.8)	1.9 (3.0)	1.2 (2.8)	1.1 (2.8)	1.0 (2.7)
Diabetes (per RxRisk)	6,492 (13%)	30,609 (12%)	3,777 (14%)	17,055 (12%)	1,810 (11%)	9,786 (12%)	3,013 (13%)	15,027 (13%)	1,868 (20%)	5,635 (12%)
MHT (b)										
Non-user	35,769 (70%)	188,007 (73%)	21,501 (77%)	105,356 (76%)	11,821 (72%)	61,212 (74%)	17,929 (76%)	86,920 (74%)	7,576 (81%)	34,036 (73%)
Oestrogen only	9,642 (19%)	49,525 (19%)	4,698 (17%)	25,889 (19%)	3,515 (21%)	15,826 (19%)	3,920 (17%)	22,794 (19%)	1,183 (13%)	9,061 (19%)
Oestrogen & progestogen	5,932 (12%)	19,108 (7%)	1,571 (6%)	7,558 (5%)	1,158 (7%)	5,407 (7%)	1,646 (7%)	7,738 (7%)	582 (6%)	3,597 (8%)

	Thyroid Cancer		Pancreatic Cancer		Kidney Cancer		Cervical Cancer		Stomach Cancer	
**Category**	Case	Control	Case	Control	Case	Case	Control	Case	Control	Case
Total	N=3,064	N=15,315	N=7,641	N=38,190	N=4,685	N=23,422	N=1,733	N=8,665	N=4,113	N=20,562
Osteoporosis medications (a)										
Non-user	2,629 (86%)	13,169 (86%)	5,941 (78%)	29,810 (78%)	3,868 (83%)	19,253 (82%)	1,491 (86%)	7,271 (84%)	3,209 (78%)	16,176 (79%)
NBB only	356 (12%)	1,708 (11%)	1,370 (18%)	6,711 (18%)	652 (14%)	3,287 (14%)	197 (11%)	1,106 (13%)	732 (18%)	3,523 (17%)
Raloxifene only	20 (1%)	138 (1%)	93 (1%)	532 (1%)	54 (1%)	265 (1%)	15 (1%)	83 (1%)	48 (1%)	261 (1%)
nNBB only	<6	<25	<15	75 (0%)	<6	31 (0%)	<6	16 (0%)	<10	37 (0%)
Strontium &/or denosumab only	<10	61 (0%)	49 (1%)	238 (1%)	16 (0%)	122 (1%)	<6	44 (1%)	20 (0%)	107 (1%)
NBB and other osteo medication/s	48 (2%)	214 (1%)	173 (2%)	791 (2%)	90 (2%)	444 (2%)	24 (1%)	138 (2%)	96 (2%)	441 (2%)
>1 osteo medication, excluding NBB	0 (0%)	<6	<6	33 (0%)	<6	20 (0%)	0 (0%)	7 (0%)	<6	17 (0%)
Weighted RxRisk V Score (mean(SD))	1.2 (2.7)	1.0 (2.7)	1.8 (3.0)	1.3 (2.9)	1.5 (3.0)	1.2 (2.8)	1.2 (2.7)	1.2 (2.8)	1.6 (3.0)	1.4 (2.9)
Diabetes (per RxRisk)	433 (14%)	1,904 (12%)	1,648 (22%)	4,719 (12%)	832 (18%)	3,013 (13%)	246 (14%)	1,079 (12%)	700 (17%)	2,566 (12%)
MHT (b)										
Non-user	2,174 (71%)	11,227 (73%)	5,861 (77%)	29,246 (77%)	3,478 (74%)	17,322 (74%)	1,495 (86%)	6,476 (75%)	3,279 (80%)	15,785 (77%)
Oestrogen only	641 (21%)	2,792 (18%)	1,406 (18%)	6,971 (18%)	899 (19%)	4,518 (19%)	145 (8%)	1,557 (18%)	654 (16%)	3,706 (18%)
Oestrogen & progestogen	249 (8%)	1,296 (8%)	374 (5%)	1,973 (5%)	308 (7%)	1,582 (7%)	93 (5%)	632 (7%)	180 (4%)	1,071 (5%)

	Liver Cancer		Brain Cancer		Bladder Cancer		Gallbladder Cancer	
**Category**	Case	Control	Case	Control	Case	Case	Control	Case
Total	N=2,434	N=12,167	N=2,613	N=13,061	N=3,731	N=18,655	N=2,387	N=11,932
Osteoporosis medications (a)								
Non-user	1,876 (77%)	9,626 (79%)	2,152 (82%)	10,730 (82%)	2,857 (77%)	14,243 (76%)	1,858 (78%)	9,273 (78%)
NBB only	446 (18%)	2,048 (17%)	375 (14%)	1,842 (14%)	690 (18%)	3,478 (19%)	427 (18%)	2,127 (18%)
Raloxifene only	37 (2%)	138 (1%)	24 (1%)	150 (1%)	58 (2%)	318 (2%)	36 (2%)	199 (2%)
nNBB only	<6	22 (0%)	<6	17 (0%)	<15	51 (0%)	<6	23 (0%)
Strontium &/or denosumab only	19 (1%)	82 (1%)	<15	64 (0%)	17 (0%)	120 (1%)	15 (1%)	65 (1%)
NBB and other osteo medication/s	48 (2%)	240 (2%)	48 (2%)	243 (2%)	97 (3%)	437 (2%)	45 (2%)	233 (2%)
>1 osteo medication, excluding NBB	<6	11 (0%)	<6	15 (0%)	<6	8 (0%)	<6	12 (0%)
Weighted RxRisk V Score (mean(sd))	1.9 (3.0)	1.3 (2.9)	1.1 (2.8)	1.1 (2.8)	1.7 (3.1)	1.5 (3.0)	1.7 (3.0)	1.4 (2.9)
Diabetes (per RxRisk)	644 (26%)	1,604 (13%)	305 (12%)	1,668 (13%)	538 (14%)	2,256 (12%)	437 (18%)	1,527 (13%)
MHT (b)								
Non-user	1,910 (78%)	9,353 (77%)	1,950 (75%)	9,615 (74%)	2,923 (78%)	14,392 (77%)	1,909 (80%)	9,108 (76%)
Oestrogen only	410 (17%)	2,158 (18%)	507 (19%)	2,568 (20%)	634 (17%)	3,349 (18%)	374 (16%)	2,229 (19%)
Oestrogen & progestogen	114 (5%)	656 (5%)	156 (6%)	878 (7%)	174 (5%)	914 (5%)	104 (4%)	595 (5%)

NBB: nitrogen-based bisphosphonates; nNBB: non-nitrogen-based bisphosphonates; PBS: Pharmaceutical Benefits Scheme; SD: standard deviation;

[a] Exclusive use of each osteoporosis medication type or combined medications

[b] Menopause Hormone Therapy, use=2 or more scripts in 12 months

Supplementary Table 9: Association between osteoporosis medication use and cancers, adjusting for diabetes medicines and menopausal hormone therapy in the concessional dataset

Cancer site	Osteoporosis medication	Cases n	Cases %	Controls n	Controls %	OR[a]	95% CI	OR[b]	95% CI	OR[c]	95% CI	OR[d]	95% CI
Breast	No osteoporosis medicine use	44,163	86	214,636	84	Reference		Reference		Reference		Reference	
	NBB[e]	5,888	11	33,344	13	0.84	0.82,0.87	0.84	0.82,0.87	0.84	0.82,0.87	0.84	0.81,0.87
	Raloxifene[e]	340	1	2,604	1	0.63	0.56,0.70	0.62	0.56,0.70	0.63	0.56,0.70	0.63	0.56,0.70
Colorectal	No osteoporosis medicine use	21,970	79	109,480	79	Reference		Reference		Reference		Reference	
	NBB[e]	4,685	17	23,291	17	1.00	0.97,1.04	0.99	0.96,1.03	1.00	0.96,1.04	1.00	0.97,1.04
	Raloxifene[e]	335	1	1,851	1	0.90	0.80,1.01	0.90	0.80,1.01	0.90	0.80,1.01	0.90	0.80,1.01
Melanoma	No osteoporosis medicine use	13,440	81	66,911	81	Reference		Reference		Reference		Reference	
	NBB[e]	2,471	15	12,245	15	1.00	0.96,1.05	1.00	0.95,1.05	0.99	0.94,1.04	0.99	0.94,1.04
	Raloxifene[e]	185	1	976	1	0.94	0.80,1.10	0.94	0.80,1.10	0.93	0.80,1.09	0.94	0.80,1.10
Lung	No osteoporosis medicine use	18,352	78	95,458	81	Reference		Reference		Reference		Reference	
	NBB[e]	4,083	17	17,502	15	1.23	1.19,1.28	1.16	1.11,1.20	1.14	1.10,1.19	1.14	1.10,1.19
	Raloxifene[e]	322	1	1,420	1	1.19	1.06,1.35	1.14	1.01,1.29	1.12	0.99,1.27	1.12	0.99,1.27
Uterus	No osteoporosis medicine use	8,388	90	39,262	84	Reference		Reference		Reference		Reference	
	NBB[e]	753	8	5,943	13	0.57	0.53,0.62	0.57	0.53,0.62	0.59	0.55,0.65	0.60	0.55,0.65
	Raloxifene[e]	75	1	473	1	0.72	0.56,0.92	0.72	0.56,0.92	0.77	0.60,0.99	0.77	0.60,0.99
Thyroid	No osteoporosis medicine use	2,629	86	13,169	86	Reference		Reference		Reference		Reference	
	NBB[e]	356	12	1,708	11	1.05	0.92,1.19	1.03	0.91,1.17	1.03	0.91,1.17	1.03	0.91,1.17
	Raloxifene[e]	20	1	138	1	0.73	0.45,1.17	0.72	0.45,1.16	0.72	0.45,1.16	0.73	0.45,1.16
Pancreas	No osteoporosis medicine use	5,941	78	29,810	78	Reference		Reference		Reference		Reference	
	NBB[e]	1,370	18	6,711	18	1.03	0.96,1.10	0.98	0.92,1.05	1.02	0.95,1.09	1.02	0.95,1.09
	Raloxifene[e]	93	1	532	1	0.88	0.70,1.10	0.84	0.67,1.04	0.87	0.69,1.08	0.87	0.69,1.08
Kidney	No osteoporosis medicine use	3,868	83	19,253	82	Reference		Reference		Reference		Reference	
	NBB[e]	652	14	3,287	14	0.99	0.90,1.08	0.96	0.87,1.05	0.97	0.88,1.07	0.97	0.88,1.07
	Raloxifene[e]	54	1	265	1	1.01	0.75,1.36	1.00	0.74,1.34	1.02	0.76,1.38	1.02	0.76,1.38
Cervix	No osteoporosis medicine use	1,491	86	7,271	84	Reference		Reference		Reference		Reference	
	NBB[e]	197	11	1,106	13	0.85	0.72,1.01	0.86	0.72,1.01	0.86	0.73,1.02	0.87	0.74,1.04
	Raloxifene[e]	15	1	83	1	0.87	0.50,1.52	0.88	0.50,1.53	0.88	0.51,1.54	0.85	0.49,1.49
Stomach	No osteoporosis medicine use	3,209	78	16,176	79	Reference		Reference		Reference		Reference	
	NBB[e]	732	18	3,523	17	1.05	0.96,1.15	1.03	0.94,1.12	1.04	0.95,1.14	1.05	0.95,1.15
	Raloxifene[e]	48	1	261	1	0.93	0.68,1.27	0.91	0.67,1.24	0.92	0.67,1.25	0.92	0.67,1.25
Liver	No osteoporosis medicine use	1,876	77	9,626	79	Reference		Reference		Reference		Reference	
	NBB[e]	446	18	2,048	17	1.13	1.00,1.27	1.06	0.94,1.20	1.11	0.98,1.25	1.11	0.99,1.25

	Raloxifene[e]	37	2	138	1	1.39	0.96,2.00	1.33	0.92,1.93	1.39	0.95,2.01	1.38	0.95,2.01
Brain	No osteoporosis medicine use	2,152	82	10,730	82	Reference		Reference		Reference		Reference	
	NBB[e]	375	14	1,842	14	1.02	0.90,1.15	1.02	0.90,1.16	1.01	0.90,1.15	1.01	0.90,1.15
	Raloxifene[e]	24	1	150	1	0.80	0.52,1.23	0.80	0.52,1.23	0.79	0.51,1.23	0.80	0.51,1.23
Bladder	No osteoporosis medicine use	2,857	77	14,243	76	Reference		Reference		Reference		Reference	
	NBB[e]	690	18	3,478	19	0.99	0.90,1.09	0.97	0.88,1.06	0.97	0.89,1.07	0.98	0.89,1.07
	Raloxifene[e]	58	2	318	2	0.91	0.68,1.21	0.89	0.67,1.19	0.90	0.68,1.20	0.90	0.68,1.20
Gallbladder	No osteoporosis medicine use	1,858	78	9,273	78	Reference		Reference		Reference		Reference	
	NBB[e]	427	18	2,127	18	1.00	0.89,1.13	0.97	0.86,1.09	0.99	0.88,1.12	0.99	0.88,1.12
	Raloxifene[e]	36	2	199	2	0.90	0.63,1.29	0.87	0.60,1.24	0.89	0.62,1.28	0.89	0.62,1.28

NBB: nitrogen-based bisphosphonates; nNBB: non-nitrogen-based bisphosphonates OR: odds ratio; CI: confidence interval

[a] All models matched by age, SEIFA, remoteness and registered state.

[b] Adjusted for RxRisk

[c] Adjusted for weighted RxRisk score and diabetes medication

[d] Adjusted for weighted RxRisk score, diabetes medication and MHT use

[e] Exclusive use of either NBB or raloxifene, no use of other osteoporosis medicines.

Supplementary Table 10: Association between duration of exclusive NBB use and cancers, concessional dataset

Cancer site	Osteoporosis medication	Cases		Controls		Total	OR	95% CI
		n	%	n	%	n		
Breast	No osteoporosis medicine use	26,440	85	128,221	82	154,661	Reference	
	<1y NBB use [c]	873	3	4,552	3	5,425	0.91	0.85,0.98
	1- <3y NBB use [c,d]	898	3	5,168	3	6,066	0.83	0.77,0.89
	3- <5y NBB use [c,e]	764	2	4,620	3	5,384	0.79	0.73,0.85
	>=5y NBB use [c, f]	1,317	4	7,447	5	8,764	0.84	0.79,0.89
Colorectal	No osteoporosis medicine use	12,568	77	62,183	76	74,751	Reference	
	<1y NBB use [c]	611	4	3,026	4	3,637	0.99	0.90,1.08
	1- <3y NBB use [c,d]	699	4	3,419	4	4,118	1.00	0.92,1.09
	3- <5y NBB use [c,e]	599	4	3,146	4	3,745	0.93	0.85,1.02
	>=5y NBB use [c, f]	1,038	6	5,332	7	6,370	0.95	0.89,1.02
Melanoma	No osteoporosis medicine use	7,917	80	39,302	79	47,219	Reference	
	<1y NBB use [c]	334	3	1,698	3	2,032	0.96	0.86,1.09
	1- <3y NBB use [c,d]	371	4	1,865	4	2,236	0.98	0.87,1.10
	3- <5y NBB use [c,e]	329	3	1,667	3	1,996	0.97	0.86,1.09
	>=5y NBB use [c, f]	573	6	2,775	6	3,348	1.02	0.92,1.12
Lung	No osteoporosis medicine use	10,894	76	57,217	79	68,111	Reference	
	<1y NBB use [c]	605	4	2,308	3	2,913	1.31	1.19,1.44
	1- <3y NBB use [c,d]	718	5	2,700	4	3,418	1.32	1.21,1.44
	3- <5y NBB use [c,e]	549	4	2,473	3	3,022	1.12	1.01,1.23
	>=5y NBB use [c, f]	893	6	4,032	6	4,925	1.12	1.04,1.21
Uterus	No osteoporosis medicine use	5,092	89	23,644	83	28,736	Reference	
	<1y NBB use [c]	107	2	820	3	927	0.59	0.48,0.72
	1- <3y NBB use [c,d]	135	2	908	3	1,043	0.67	0.55,0.80
	3- <5y NBB use [c,e]	117	2	845	3	962	0.62	0.51,0.75
	>=5y NBB use [c, f]	125	2	1,336	5	1,461	0.41	0.34,0.49
Thyroid	No osteoporosis medicine use	1,665	85	8,382	85	10,047	Reference	
	<1y NBB use [c]	60	3	270	3	330	1.10	0.83,1.47
	1- <3y NBB use [c,d]	57	3	273	3	330	1.03	0.77,1.39
	3- <5y NBB use [c,e]	48	2	223	2	271	1.07	0.78,1.47
	>=5y NBB use [c, f]	70	4	342	3	412	1.01	0.77,1.32
Pancreas	No osteoporosis medicine use	3,453	75	17,301	76	20,754	Reference	
	<1y NBB use [c]	170	4	821	4	991	1.00	0.84,1.18
	1- <3y NBB use [c,d]	203	4	1,027	4	1,230	0.95	0.81,1.11
	3- <5y NBB use [c,e]	188	4	932	4	1,120	0.98	0.83,1.15
	>=5y NBB use [c, f]	324	7	1,607	7	1,931	0.98	0.86,1.11
Kidney	No osteoporosis medicine use	2,328	81	11,565	80	13,893	Reference	
	<1y NBB use [c]	94	3	439	3	533	1.03	0.82,1.30
	1- <3y NBB use [c,d]	109	4	499	3	608	1.05	0.84,1.29
	3- <5y NBB use [c,e]	83	3	469	3	552	0.86	0.67,1.09
	>=5y NBB use [c, f]	146	5	747	5	893	0.94	0.78,1.13
Cervix	No osteoporosis medicine use	859	85	4,181	82	5,040	Reference	
	<1y NBB use [c]	42	4	149	3	191	1.35	0.94,1.93
	1- <3y NBB use [c,d]	28	3	173	3	201	0.77	0.51,1.17
	3- <5y NBB use [c,e]	23	2	146	3	169	0.76	0.48,1.19
	>=5y NBB use [c, f]	35	3	224	4	259	0.74	0.51,1.08
Stomach	No osteoporosis medicine use	1,782	76	8,924	76	10,706	Reference	
	<1y NBB use [c]	94	4	432	4	526	1.06	0.85,1.34
	1- <3y NBB use [c,d]	91	4	516	4	607	0.86	0.68,1.09
	3- <5y NBB use [c,e]	97	4	454	4	551	1.04	0.83,1.31
	>=5y NBB use [c, f]	162	7	764	7	926	1.04	0.87,1.25
Liver	No osteoporosis medicine use	1,118	74	5,841	77	6,959	Reference	
	<1y NBB use [c]	72	5	273	4	345	1.30	1.00,1.71

	1- <3y NBB use [c,d]	67	4	323	4	390	1.04	0.79,1.37
	3- <5y NBB use [c,e]	50	3	282	4	332	0.89	0.65,1.21
	>=5y NBB use [c, f]	118	8	478	6	596	1.23	0.99,1.53
Brain	No osteoporosis medicine use	1,279	81	6,322	80	7,601	Reference	
	<1y NBB use [c]	59	4	244	3	303	1.20	0.89,1.60
	1- <3y NBB use [c,d]	49	3	298	4	347	0.81	0.60,1.11
	3- <5y NBB use [c,e]	47	3	242	3	289	0.96	0.69,1.32
	>=5y NBB use [c, f]	83	5	405	5	488	1.01	0.79,1.30
Bladder	No osteoporosis medicine use	1,620	75	7,905	73	9,525	Reference	
	<1y NBB use [c]	68	3	412	4	480	0.78	0.60,1.02
	1- <3y NBB use [c,d]	103	5	553	5	656	0.88	0.71,1.10
	3- <5y NBB use [c,e]	92	4	490	5	582	0.89	0.71,1.13
	>=5y NBB use [c, f]	155	7	833	8	988	0.89	0.74,1.06
Gallbladder	No osteoporosis medicine use	1,111	75	5,556	75	6,667	Reference	
	<1y NBB use [c]	61	4	266	4	327	1.10	0.83,1.47
	1- <3y NBB use [c,d]	64	4	333	5	397	0.93	0.70,1.22
	3- <5y NBB use [c,e]	57	4	297	4	354	0.93	0.69,1.24
	>=5y NBB use [c, f]	100	7	545	7	645	0.89	0.71,1.11

CI: confidence interval; NBB: nitrogen-based bisphosphonates; OR: odds ratio; y: years.

[a] All models matched by age, SEIFA, remoteness and registered state, and adjusted for weighted RxRisk score.

[b] Excludes cases diagnosed prior to 1st July 2008.

[c] No use of other osteoporosis medicines.

[d] Minimum 1 year duration of use plus a minimum 292 defined daily doses.

[e] Minimum 3 years duration of use plus a minimum 877 defined daily doses.

[f] Minimum 5 years.

Supplementary Table 11: Characteristics of women diagnosed with cancer in Western Australian

	Breast	Colorectal	Melanoma	Lung	Uterus	Thyroid	Pancreas	Kidney	Cervix	Stomach	Liver	Brain	Bladder	Gallbladder
Total Cases	N=8,218	N=2,904	N=2,335	N=2,665	N=1,173	N=576	N=824	N=540	N=237	N=403	N=219	N=362	N=349	N=252
Index age (y), mean (SD)	65.9 (10.9)	72.6 (11.4)	68.1 (11.6)	71.9 (10.4)	66.9 (10.3)	62.2 (9.5)	74.3 (11.1)	69.0 (10.8)	65.7 (11.9)	73.8 (11.4)	72.8 (12.0)	69.7 (10.9)	76.6 (11.2)	75.3 (10.3)
Index age (y)														
50-59y	2,915 (35%)	482 (17%)	726 (31%)	410 (15%)	358 (31%)	289 (50%)	96 (12%)	126 (23%)	94 (40%)	67 (17%)	42 (19%)	86 (24%)	35 (10%)	20 (8%)
60-69y	2,733 (33%)	720 (25%)	642 (27%)	730 (27%)	408 (35%)	173 (30%)	196 (24%)	174 (32%)	63 (27%)	74 (18%)	45 (21%)	93 (26%)	57 (16%)	59 (23%)
70-79y	1,469 (18%)	818 (28%)	532 (23%)	887 (33%)	253 (22%)	78 (14%)	249 (30%)	141 (26%)	44 (19%)	136 (34%)	65 (30%)	110 (30%)	109 (31%)	87 (35%)
80-89y	919 (11%)	722 (25%)	353 (15%)	549 (21%)	130 (11%)	30 (5%)	224 (27%)	84 (16%)	27 (11%)	99 (25%)	54 (25%)	65 (18%)	107 (31%)	69 (27%)
>=90y	182 (2%)	162 (6%)	82 (4%)	89 (3%)	24 (2%)	6 (1%)	59 (7%)	15 (3%)	9 (4%)	27 (7%)	13 (6%)	8 (2%)	41 (12%)	17 (7%)
SEIFA														
1 (most disadvantaged)	1,390 (17%)	573 (20%)	413 (18%)	571 (21%)	216 (18%)	113 (20%)	167 (20%)	119 (22%)	55 (23%)	84 (21%)	48 (22%)	50 (14%)	73 (21%)	48 (19%)
2	1,949 (24%)	748 (26%)	555 (24%)	716 (27%)	312 (27%)	157 (27%)	199 (24%)	134 (25%)	51 (22%)	98 (24%)	42 (19%)	103 (28%)	86 (25%)	84 (33%)
3	1,563 (19%)	511 (18%)	423 (18%)	488 (18%)	246 (21%)	100 (17%)	158 (19%)	107 (20%)	47 (20%)	73 (18%)	48 (22%)	68 (19%)	61 (17%)	47 (19%)
4	1,621 (20%)	544 (19%)	443 (19%)	491 (18%)	215 (18%)	117 (20%)	153 (19%)	93 (17%)	49 (21%)	80 (20%)	34 (16%)	62 (17%)	68 (19%)	45 (18%)
5 (least disadvantaged)	1,695 (21%)	528 (18%)	501 (21%)	399 (15%)	184 (16%)	89 (15%)	147 (18%)	87 (16%)	35 (15%)	68 (17%)	47 (21%)	79 (22%)	61 (17%)	28 (11%)
Remoteness														
Major City	6,030 (73%)	2,056 (71%)	1,599 (68%)	1,940 (73%)	836 (71%)	412 (72%)	592 (72%)	384 (71%)	174 (73%)	310 (77%)	174 (79%)	269 (74%)	259 (74%)	170 (67%)
Inner Regional	1,116 (14%)	434 (15%)	391 (17%)	372 (14%)	162 (14%)	82 (14%)	137 (17%)	98 (18%)	25 (11%)	53 (13%)	19 (9%)	42 (12%)	43 (12%)	46 (18%)
Outer Regional	777 (9%)	306 (11%)	237 (10%)	241 (9%)	117 (10%)	55 (10%)	72 (9%)	39 (7%)	24 (10%)	32 (8%)	17 (8%)	41 (11%)	36 (10%)	23 (9%)
Remote & Very Remote	295 (4%)	108 (4%)	108 (5%)	112 (4%)	58 (5%)	27 (5%)	23 (3%)	19 (4%)	14 (6%)	8 (2%)	9 (4%)	10 (3%)	11 (3%)	13 (5%)

SD: standard deviation; SEIFA: Socio-Economic Indexes for Areas; y: years.

Supplementary Table 12: Characteristics of women diagnosed with cancer, medication use from PBS, WA data

	Breast		Colorectal		Lung		Uterus		Thyroid		Cervix	
	Case	Control	Case	Control	Case	Control	Case	Control	Case	Control	Case	Control
Total	N=8,218	N=41,086	N=2,904	N=14,520	N=2,665	N=13,325	N=1,173	N=5,865	N=576	N=2,880	N=237	N=1,185
Osteoporosis medications (a)												
Non-user	7,602 (93%)	37,470 (91%)	2,494 (86%)	12,385 (85%)	2,243 (84%)	11,435 (86%)	1,091 (93%)	5,310 (91%)	547 (95%)	2,737 (95%)	219 (92%)	1,078 (91%)
NBB only	514 (6%)	2,893 (7%)	326 (11%)	1,709 (12%)	328 (12%)	1,497 (11%)	61 (5%)	454 (8%)	<30	111 (4%)	<20	87 (7%)
Raloxifene only	29 (0%)	235 (1%)	23 (1%)	114 (1%)	27 (1%)	115 (1%)	<10	27 (0%)	0 (0%)	<15	<6	<10
nNBB only	<6	31 (0%)	<6	<20	<6	17 (0%)	0 (0%)	<6	0 (0%)	<6	0 (0%)	0 (0%)
Strontium &/or denosumab only	<20	96 (0%)	<20	65 (0%)	<15	53 (0%)	<6	<15	0 (0%)	<6	<6	<6
NBB and other osteo medication/s	51 (1%)	350 (1%)	41 (1%)	228 (2%)	50 (2%)	198 (1%)	10 (1%)	59 (1%)	<6	15 (1%)	<6	11 (1%)
>1 osteo medication, excluding NBB	<6	11 (0%)	<6	<6	<6	10 (0%)	0 (0%)	0 (0%)	0 (0%)	0 (0%)	0 (0%)	0 (0%)
Weighted RxRisk V Score (mean(SD))	0.5 (2.2)	0.5 (2.2)	0.9 (2.5)	0.9 (2.6)	1.5 (2.9)	0.9 (2.5)	0.6 (2.3)	0.6 (2.3)	0.6 (2.2)	0.4 (2.1)	0.6 (2.1)	0.6 (2.2)
Diabetes (per RxRisk)	662 (8%)	3,046 (7%)	332 (11%)	1,357 (9%)	304 (11%)	1,333 (10%)	161 (14%)	497 (8%)	56 (10%)	215 (7%)	21 (9%)	89 (8%)
MHT (b)												
Non-user	6,568 (80%)	33,309 (81%)	2,330 (80%)	11,420 (79%)	2,099 (79%)	10,367 (78%)	1,029 (88%)	4,656 (79%)	468 (81%)	2,383 (83%)	219 (92%)	963 (81%)
Oestrogen only	971 (12%)	5,432 (13%)	389 (13%)	2,302 (16%)	364 (14%)	2,124 (16%)	90 (8%)	831 (14%)	66 (11%)	328 (11%)	10 (4%)	146 (12%)
Oestrogen & progestogen	679 (8%)	2,345 (6%)	185 (6%)	798 (5%)	202 (8%)	834 (6%)	54 (5%)	378 (6%)	42 (7%)	169 (6%)	8 (3%)	76 (6%)
Total births (c)												
No recorded births	3,368 (41%)	17,262 (42%)	1,361 (47%)	6,905 (48%)	1,211 (45%)	6,243 (47%)	495 (42%)	2,548 (43%)	231 (40%)	1,115 (39%)	107 (45%)	534 (45%)
1 recorded birth	781 (10%)	3,868 (9%)	334 (12%)	1,438 (10%)	285 (11%)	1,268 (10%)	112 (10%)	537 (9%)	44 (8%)	242 (8%)	25 (11%)	104 (9%)
2 recorded births	1,903 (23%)	8,570 (21%)	439 (15%)	2,426 (17%)	470 (18%)	2,243 (17%)	262 (22%)	1,174 (20%)	114 (20%)	656 (23%)	40 (17%)	210 (18%)
3 recorded births	1,311 (16%)	6,616 (16%)	394 (14%)	1,974 (14%)	353 (13%)	1,974 (15%)	180 (15%)	952 (16%)	116 (20%)	529 (18%)	36 (15%)	188 (16%)
4 or more recorded births	855 (10%)	4,770 (12%)	376 (13%)	1,777 (12%)	346 (13%)	1,597 (12%)	124 (11%)	654 (11%)	71 (12%)	338 (12%)	29 (12%)	149 (13%)
Bilateral oophorectomy (or >1 unilateral) (d)												
No record	7,846 (95%)	39,322 (96%)	2,800 (96%)	13,984 (96%)	2,554 (96%)	12,786 (96%)	1,151 (98%)	5,570 (95%)	537 (93%)	2,777 (96%)	237 (100%)	1,139 (96%)
Hospital record of procedure	372 (5%)	1,764 (4%)	104 (4%)	536 (4%)	111 (4%)	539 (4%)	22 (2%)	295 (5%)	39 (7%)	103 (4%)	0 (0%)	46 (4%)
Osteoporosis/pathological fracture (e)												
No record	7,773 (95%)	38,548 (94%)	2,624 (90%)	13,041 (90%)	2,369 (89%)	12,069 (91%)	1,130 (96%)	5,482 (93%)	557 (97%)	2,756 (96%)	225 (95%)	1,113 (94%)
Hospital record of diagnosis	445 (5%)	2,538 (6%)	280 (10%)	1,479 (10%)	296 (11%)	1,256 (9%)	43 (4%)	383 (7%)	19 (3%)	124 (4%)	12 (5%)	72 (6%)

MHT: menopausal hormone therapy; NBB: nitrogen-based bisphosphonate; nNBB: non-nitrogen-based bisphosphonate; PBS: Pharmaceutical Benefits Scheme; SD: standard deviation.

a Exclusive use of each osteoporosis medication type or combined medications

b Menopause Hormone Therapy, use=2 or more scripts in 12 months

c From available midwife and birth records

d From available hospital record prior to 6 months before index date

e From available hospital

Supplementary Table 13: Characteristics of women diagnosed with cancer, medication use from PBS, WA data (controls have no prior hysterectomy)

	Uterus		Cervix	
	Case	Control	Case	Control
Total	N=1,173	N=5,865	N=237	N=1,185
Osteoporosis medications (a)				
Non-user	1,091 (93%)	5,320 (91%)	219 (92%)	1,080 (91%)
NBB only	61 (5%)	432 (7%)	<20	83 (7%)
Raloxifene only	<10	33 (1%)	<6	13 (1%)
nNBB only	0 (0%)	<6	0 (0%)	0 (0%)
Strontium &/or denosumab only	<6	<25	<6	<6
NBB and other osteo medication/s	10 (1%)	55 (1%)	<6	<10
>1 osteo medication, excluding NBB	0 (0%)	<6	0 (0%)	0 (0%)
Weighted RxRisk V Score (mean(SD))	0.6 (2.3)	0.5 (2.2)	0.6 (2.1)	0.5 (2.1)
Diabetes (per RxRisk)	161 (14%)	445 (8%)	21 (9%)	78 (7%)
MHT (b)				
Non-user	1,029 (88%)	4,892 (83%)	219 (92%)	1,007 (85%)
Oestrogen only	90 (8%)	551 (9%)	10 (4%)	101 (9%)
Oestrogen & progestogen	54 (5%)	422 (7%)	8 (3%)	77 (6%)
Total births (c)				
No recorded births	495 (42%)	2,648 (45%)	107 (45%)	565 (48%)
1 recorded birth	112 (10%)	542 (9%)	25 (11%)	133 (11%)
2 recorded births	262 (22%)	1,149 (20%)	40 (17%)	218 (18%)
3 recorded births	180 (15%)	869 (15%)	36 (15%)	152 (13%)
4 or more recorded births	124 (11%)	657 (11%)	29 (12%)	117 (10%)
Bilateral oophorectomy (or >1 unilateral) (d)				
No record	1,151 (98%)	5,833 (99%)	237 (100%)	
Hospital record of procedure	22 (2%)	32 (1%)	0 (0%)	<6
Osteoporosis/pathological fracture (e)				
No record	1,130 (96%)	5,490 (94%)	225 (95%)	1,105 (93%)
Hospital record of diagnosis	43 (4%)	375 (6%)	12 (5%)	80 (7%)

NBB: nitrogen-based bisphosphonate; PBS: Pharmaceutical Benefits Scheme; SD: standard deviation.
a Exclusive use of each osteoporosis medication type or combined medications
b Menopause Hormone Therapy, use=2 or more scripts in 12 months
c From available midwife and birth records
d From available hospital record prior to 6 months before index date
e From available hospital records between 10 years and 6m months prior to index date

Supplementary Table 14: Association between nitrogen-based bisphosphonates and raloxifene and cancers, WA data, adjusted for Rx-Risk scores and covariates from health records

Cancer stie	Osteoporosis medication	Cases n	%	Controls n	%	OR	95% CI	OR [a]	95% CI	OR [b]	95% CI	OR [c]	95% CI
Breast	No osteoporosis medicine use	7,602	93	37,470	91	Reference		Reference		Reference		Reference	
	NBB[d]	514	6	2,893	7	0.86	0.78,0.95	0.85	0.77,0.95	0.85	0.77,0.95	0.86	0.78,0.96
	Raloxifene[d]	29	0	235	1	0.60	0.40,0.88	0.62	0.42,0.92	0.62	0.42,0.92	0.63	0.42,0.93
Colorectal	No osteoporosis medicine use	2,494	86	12,385	85	Reference		Reference		Reference		Reference	
	NBB[d]	326	11	1,709	12	0.94	0.83,1.08	0.94	0.82,1.07	0.94	0.82,1.07	0.96	0.83,1.10
	Raloxifene[d]	23	1	114	1	1.00	0.64,1.57	1.02	0.65,1.60	1.02	0.65,1.60	1.04	0.66,1.63
Uterus	No osteoporosis medicine use	1,091	93	5,310	91	Reference		Reference		Reference		Reference	
	NBB[d]	61	5	454	8	0.63	0.47,0.84	0.63	0.47,0.84	0.63	0.47,0.84	0.71	0.52,0.95
	Raloxifene[d]	<10		27	0	1.37	0.62,3.03	1.12	0.49,2.58	1.08	0.47,2.49	1.35	0.58,3.15
Uterus (No hysterectomy)[e]	No osteoporosis medicine use	1,091	93	5,320	91	Reference		Reference		Reference		Reference	
	NBB[d]	61	5	432	7	0.67	0.50,0.89	0.66	0.49,0.88	0.66	0.50,0.88	0.74	0.55,0.99
	Raloxifene[d]	<10		33	1	1.16	0.53,2.52	1.13	0.52,2.48	1.14	0.52,2.49	1.29	0.58,2.85
Cervix	No osteoporosis medicine use	219	92	1,078	91	Reference		Reference		Reference		Reference	
	NBB[d]	15	6	87	7	0.83	0.45,1.53	0.82	0.44,1.54	0.83	0.44,1.57	0.85	0.44,1.66
	Raloxifene[d]	<6	0	<10	1	0.70	0.08,5.93	0.72	0.08,6.42	0.72	0.08,6.49	0.73	0.08,6.53
Cervix (No hysterectomy)[e]	No osteoporosis medicine use	219	92	1,080	91	Reference		Reference		Reference		Reference	
	NBB[d]	<20		83	7	0.85	0.46,1.56	0.88	0.47,1.64	0.87	0.46,1.62	0.94	0.49,1.80
	Raloxifene[d]	<6		13	1	0.36	0.05,2.80	0.33	0.04,2.53	0.33	0.04,2.55	0.37	0.05,2.88

CI: confidence interval; NBB: nitrogen-based bisphosphonates.
All models matched by age, SEIFA, remoteness and registered state.
[a] Adjusted for weighted RxRisk
[b] Adjusted for weighted RxRisk and binary/categorical variables for parity and bilateral oophorectomy (>6m before index) from health records
[c] Adjusted for weighted RxRisk and binary/categorical variables for parity, bilateral oophorectomy (>6m before index) and osteoporosis or osteoporotic fracture from health records
[d] Exclusive use of either NBB or raloxifene, no use of other osteoporosis medicines.
[e] Women with prior hysterectomy excluded from selection as controls

Supplementary Table 15: Adjusted (margins at mean age) proportion of bisphosphonate users and non-users in each body mass index category from unpublished QSkin study data

	Bisphosphonate use	
**BMI Category**	**User**	**Non-user**
Underweight	3.4%	1.6%
Normal weight	50.5%	41.1%
Overweight	28.1%	31.0%
Obese (BMI 30-<35)	8.7%	16.9%
Obese (BMI 35-<40)	5.6%	6.2%
Obese (BMI 40+)	3.6%	3.3%
Total (excludes missing)	100%	100%

BMI: body mass index.

Supplementary Table 16: Proportion of bisphosphonate users and non-users with reported smoking status (current, former, never) from unpublished QSkin study data

	Bisphosphonate use	
**Smoking status**	**User**	**Non-user**
Current	10.7%	8.9%
Former	28.8%	30.8%
Never	60.5%	60.3%
Total (excludes missing)	100%	100%

Supplementary Table 17: Quantitative bias analysis for the effect of obesity on the association between bisphosphonate use and uterine cancer

	Prevalence for users[a]		Prevalence for non-users[a]		OR[b] for BMI mid-level	OR[b] for BMI highest-level	Crude OR[c]	SMR
	BMI mid-level	BMI highest-level	BMI mid-level	BMI highest-level				
Model 1[d]	14.3%	3.6%	23.1%	3.3%	4.45	7.14	0.63	0.74
Model 2[e]	14.3%	3.6%	23.1%	3.3%	2.15	3.11	0.63	0.68
Model 3[f]	8.7%	9.2%	16.9%	9.5%	2.52	7.14	0.63	0.69
Model 4[g]	8.7%	9.2%	16.9%	9.5%	1.73	3.11	0.63	0.67
Model 5[h]	11.5%	1.6%	23.1%	3.3%	4.89	8.06	0.66	0.87

BMI: body mass index; EC: endometrial cancer; OR: odds ratio; SMR: standardised morbidity ratio.

[a] Estimate of prevalence of obesity for bisphosphonate users and non-users (QSkin Data).

[b] Epidemiology of Endometrial Cancer Consortium (E2C2)[5] estimates for the association between BMI and risk of EC with BMI of <25 as the reference.

[c] Crude OR from 2x2 table, unconditional logistic regression for association of nitrogen-based bisphosphonate use and uterine cancer.

[d] Mid-level: Combined 30-<35 and 35-<40 BMI; Highest-level: 40+ BMI. OR for BMI 35-<40 and EC Type 1 used for mid-level, and OR for BMI 40+ and EC Type 1 for highest-level.

[e] Mid-level: Combined 30-<35 and 35-<40 BMI; Highest-level: 40+ BMI. OR for BMI 35-<40 and EC Type 2 used for mid-level, and OR for BMI 40+ and EC Type 2 for highest-level.

[f] Mid-level: 30-<35 BMI; Highest-level: combined 35-<40 and 40+ BMI. OR for BMI 30-<35 and EC Type 1 used for mid-level, and OR for BMI 40+ and EC Type 1 for highest-level.

[g] Mid-level: 30-<35 BMI; Highest-level: combined 35-<40 and 40+ BMI. OR for BMI 30-<35 and EC Type 2 used for mid-level, and OR for BMI 40+ and EC Type 2 for highest-level.

[h] Sensitivity showing results if prevalence of obesity in users was half of that in non-users. Using the upper 95% confidence intervals of the estimates for 35-<40 BMI for mid-level and 40+ BMI for highest-level.

Supplementary Table 18: Quantitative bias analysis for the effect of smoking status on the association between bisphosphonate use and lung cancer

	Prevalence for users[a]		Prevalence for non-users[a]		OR for Current Smokers and lung cancer	OR for Former Smokers and lung cancer	Crude OR[b]	SMR
	Current Smokers	Former Smokers	Current Smokers	Former Smokers				
Model 1	10.7	28.8	8.9	30.8	9.0[c]	3.9[c]	1.23	1.19
Model 2	10.7	28.8	8.9	30.8	12.1[d]	5.3[d]	1.23	1.19
Model 3	10.7	28.8	8.9	30.8	24.1[e]	3.9[e]	1.23	1.13
Model 4	17.8[f]	28.8	8.9	30.8	12.1[d]	5.3[d]	1.23	0.97

OR: odds ratio; SMR: standardised morbidity ratio.

[a] Estimate of prevalence of smoking for bisphosphonate users and non-users (QSkin Data).

[b] Crude OR from 2x2 table, unconditional logistic regression for association of nitrogen-based bisphosphonate use and lung cancer.

[c] Estimates from meta-analysis of smoking and cancer,[6] for the association between current and former smoking and risk of lung cancer, with never smokers as the reference, in men and women.

[d] Upper 95% confidence interval RR[6] between smoking status and risk of lung cancer, with never smokers as the reference, in men and women.

[e] Sensitivity using the highest estimate for association[6] in women with a cigarette consumption >=20/day for current smokers.

[f] Sensitivity using a scenario of double the number of current smokers in bisphosphonate users compared to non-users.
